# Effects of Ghrelin Treatment on Exercise Capacity in Underweight COPD Patients: a substudy of a multicenter, randomized, double-blind, placebo-controlled trial of ghrelin treatment

**DOI:** 10.1186/1471-2466-13-37

**Published:** 2013-06-10

**Authors:** Keisuke Miki, Ryoji Maekura, Noritoshi Nagaya, Seigo Kitada, Mari Miki, Kenji Yoshimura, Yoshitaka Tateishi, Masaharu Motone, Toru Hiraga, Masahide Mori, Kenji Kangawa

**Affiliations:** 1 Department of Internal medicine, National Hospital Organization Toneyama National Hospital, 5-1-1 Toneyama, Toyonaka, Osaka, 560-8552, Japan; 2 Department of Regenerative Medicine, National Cerebral and Cardiovascular Center Research Institute, Suita, Japan; 3 Department of Biochemistry, National Cerebral and Cardiovascular Center Research Institute, Suita, Japan

**Keywords:** Dyspnea, Exercise, Pulmonary rehabilitation, Sympathetic nerve activity, Underweight

## Abstract

**Background:**

The aim of this substudy of the ghrelin treatment, multicenter, randomized, double-blind, placebo-controlled trial was to investigate the effects of ghrelin administration on exercise capacity and the underlying mechanisms in underweight patients with chronic obstructive pulmonary disease (COPD) using cardiopulmonary exercise testing.

**Methods:**

Twenty underweight COPD patients were randomized to pulmonary rehabilitation with intravenous ghrelin (2 μg/kg, n = 10) or placebo (n = 10) twice daily for 3 weeks in a double-blind fashion. The primary outcome was changes in peak oxygen uptake $\left(\overset{•}{V}o_{2}\right)$. Secondary outcomes included changes in exertional cardio-respiratory functions: O_2_-pulse, physiologic dead space/tidal volume-ratio (V_D_/V_T_), ventilatory equivalent for oxygen $\left({\overset{•}{V}}_{E}/\overset{•}{V}o_{2}\right)$, and ventilatory equivalent for carbon dioxide $\left(\overset{•}{V}E/\overset{•}{V}co_{2}\right)$.

**Results:**

With incremental exercise, at peak exercise, there was a significant difference in the mean difference (ghrelin minus placebo), i.e., treatment effect in: i) peak $\overset{•}{V}o_{2}$ (1.2 mL/kg/min, 95% CI: 0.2-2.3 mL/kg/min, between-group p = 0.025); ii) $\overset{•}{V}E/\overset{•}{V}o_{2}$ (-4.2, 95% CI: -7.9 to -0.5, between-group p = 0.030); iii) $\overset{•}{V}E\overset{•}{/V}co_{2}$ (-4.1, 95% CI: -8.2 to -0.1, between-group p = 0.045); iv) V_D_/V_T_ (-0.04, 95% CI: -0.08 to -0.00, between-group p = 0.041); and v) O_2_-pulse (0.7 mL/beat, 95% CI: 0.3 to 1.2 mL/beat, between-group p = 0.003). Additionally, repeated-measures analysis of variance (ANOVA) indicated a significant time-course effect of ghrelin versus placebo in the peak $\overset{•}{V}o_{2}$ (p = 0.025).

**Conclusion:**

Ghrelin administration was associated with improved exertional capacity and improvements in ventilatory-cardiac parameters.

**Trial registration:**

UMIN (University Hospital Medical Information Network in Japan) C000000061

## Background

Chronic obstructive pulmonary disease (COPD) is a major health issue, especially in its advanced stages. It is characterized by a significant systemic deterioration, so-called pulmonary cachexia, that affects exercise capacity, quality of life, and survival in such patients [1-6]. Treatment strategies targeting pulmonary cachexia have been attempted and have provided insight into the pathogenetic mechanisms of pulmonary cachexia [2,7].

Pulmonary rehabilitation (PR) is recommended as a therapeutic strategy in many diseases, including cachectic COPD [8]. However, it has been important to determine whether cachectic COPD patients are capable of adaptive responses to PR, because PR may partially disturb anabolic and catabolic balance in such patients [2,9].

Ghrelin is an endogenous ligand for growth hormone (GH) secretagogue receptor (GHSR), originally isolated from the stomach [10]. Given that ghrelin has GH-releasing activity, ghrelin may have beneficial effects in COPD patients through a GH-dependent mechanism. Meanwhile, as a GH-independent mechanism, ghrelin induces a positive energy balance by decreasing fat utilization [11], stimulating food intake and adiposity [12], increasing cardiac output in healthy humans [13], and inhibiting sympathetic nerve activity [13,14]. In an open-label pilot study, we previously demonstrated that ghrelin may improve walking distance and inhibit sympathetic nerve activity in underweight COPD patients [15]. More recently, a multicenter, randomized, double-blind, placebo-controlled trial was conducted to investigate the efficacy and safety of adding ghrelin to PR in underweight COPD patients, and it showed that ghrelin administration improved symptoms and respiratory muscle strength. While the results were considered most likely to have been due to ghrelin treatment, the precise mechanism that underlies the improvement of exercise performance or symptoms remains unclear. This study was simultaneously conducted in a single center as part of a multicenter, randomized, double-blind, placebo-controlled study of underweight COPD patients [16]. The primary objective was to investigate whether ghrelin-PR combination treatment would improve exercise capacity. A secondary objective was to determine whether the underlying mechanisms of the improvements in response to ghrelin would be associated with improvement of exertional cardio-respiratory functions and attenuation of exertional sympathetic nervous system activity. Some results of this trial have been reported in abstract form [17].

## Methods

The protocol for this trial and Supplementary Results are available as supporting information; see Additional file 1 (Protocol) and Additional file 2 (Supplementary Results).

### Subjects

The eligibility criteria for participation were as follows: 1) moderate to severe COPD (FEV1% of less than 70% and % forced expiratory volume in one second (FEV1) of less than 50%); 2) underweight (body mass index (BMI) < 21 kg/m^2^); 3) clinically stable and able to participate in PR; 4) between 20 and 85 years old; and 5) signed the agreement for participation in this study. Participants were excluded if they met any of the following: 1) malignant tumors; 2) active infection; 3) severe heart disease; 4) hepatic dysfunction (serum levels of aspartate aminotransferase and alanine aminotransferase twice the upper limit of normal or more); 5) asthma; 6) definitely or possibly pregnant; 7) change in drug regimen within 4 weeks before participation in this study; or 8) judged to be unable to participate in this study by their physician, in addition to the above exclusion criteria.

### Study design

This study was a 3-week, randomized, double-blind, placebo-controlled trial of ghrelin administration during PR conducted at a single center (Toneyama National Hospital) as part of a main multicenter trial [16]. To test the primary hypothesis that ghrelin-PR combination treatment would improve exercise capacity, recruitment was from September 2005 until the target sample size of 18 was achieved on May 2009.

**Figure 1:**
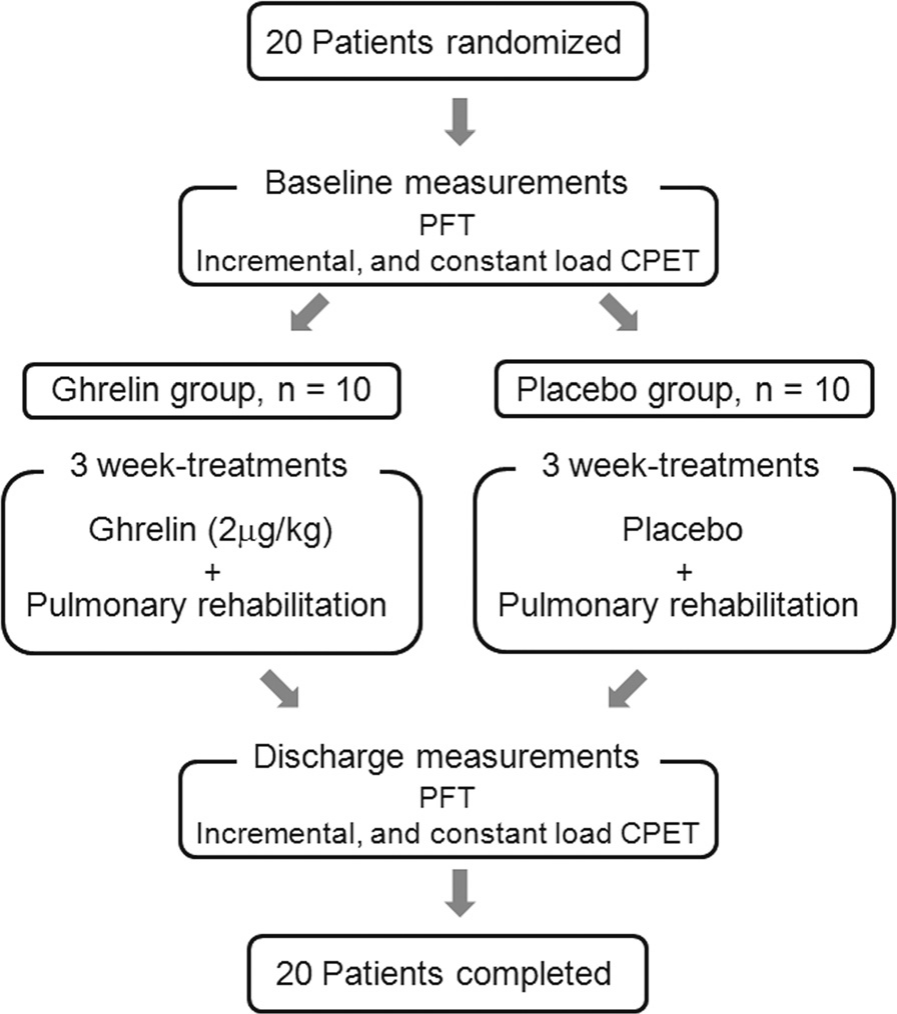
**Outline of study design.** PFT, pulmonary function test; CPET, cardiopulmonary exercise testing.

This study was approved by the ethics committee of the National Hospital Organization Toneyama National Hospital (approval number, 0311) and was conducted according to the Declaration of Helsinki and Good Clinical Practice guidelines. Written informed consent was obtained from all subjects (in Japanese). Enrolled patients were randomly assigned in a 1:1 ratio to receive 3-week PR with either ghrelin or placebo in a double-blind fashion (Figure 1). The administration of ghrelin (2 μg/kg, ghrelin solution with 10 mL saline) or placebo was done intravenously over 30 minutes at a constant rate and repeated twice daily for 3 weeks in hospital. Randomization was done in our center considered as a block in the main multicenter study [16]. The randomization list was generated by a statistician from Hamamatsu University School of Medicine and maintained there until the study was finished and unblinded. Neither the physicians nor the patients were aware of the treatment assignments. The test drug controller managed a copy of the randomization list and the test drugs at the pharmacy department of the National Hospital Organization Toneyama National Hospital. At study completion, the randomization list was used to confirm appropriate conduct of the trial. Patients were tested at pre-treatment and post-treatment, i.e., Week 3. The main multicenter study [16], including this study, is registered at https://upload.umin.ac.jp/cgi-open-bin/ctr/ctr.cgi?function=brows&action=brows&type=summary&recptno=R000000103&language=E, number C000000061.

### Preparation of human ghrelin

Human ghrelin was prepared as described previously [15,16]. In brief, ghrelin was stored in 2-mL volumes, each containing 120 μg ghrelin. The chemical nature and content of the human ghrelin in vials were confirmed as described previously [15,16]. All vials were stored frozen at -30°C until the time of preparation for administration.

### Pulmonary rehabilitation (PR)

Aerobic exercise training included in the PR program was conducted for 5 days per week, for 3 weeks, at high-intensity targets [16]. The PR program consisted of disease education and instruction of patients and their families, physical therapy, i.e., conditioning including breathing control techniques, and aerobic exercise training. Occupational therapy was not included in the present PR program. The aerobic exercise training sessions were conducted as three sets (with 10-min breaks) daily, 5 days per week, for 3 weeks, and they were performed on electromechanically braked cycle ergometers. The initial exercise level of each set was set for 6 min at the work rate corresponding to 60% of the peak $\overset{•}{V}o_{2}$attained on baseline cardiopulmonary exercise testing (CPET). As tolerated by the subject, the exercise duration was initially increased to 10 min. After that, the training work rate was increased by 5 W, and then extended to the work rate corresponding to 80% of the baseline peak $\overset{•}{V}o_{2}$. If the subject found the set intolerable, it was reduced to its previous setting. Supplemental oxygen was used if necessary to maintain an oxygen saturation >90% during exercise training.

### Outcome measures

The primary outcome was change in exercise capacity, i.e., peak oxygen uptake $\left(\overset{•}{V}o_{2}\right)$. Secondary outcomes were changes in exertional dyspnea and plasma norepinephrine levels, as well as exertional cardio-respiratory functions: O_2_-pulse ($\overset{•}{V}o_{2}/\mathrm{HR}$ i.e., the product of stroke volume and the arterial-mixed venous O_2_ difference), physiologic dead space/tidal volume ratio (V_D_/V_T_, i.e., the degree of mismatching of ventilation to perfusion), ventilatory equivalent for oxygen ($\overset{•}{V}E/\overset{•}{V}o_{2}$ , i.e., measurement of the ventilatory requirement for the metabolic rate), ventilatory equivalent for carbon dioxide ($\overset{•}{V}E\overset{•}{/V}co_{2}$, i.e., measurement of the ventilatory requirement for the metabolic rate), and inspiratory capacity (IC).

### Procedures

Pulmonary function measurements were done as previously described [18]. Symptom-limited exercise tests were conducted on an electrically-braked cycle ergometer (CV-1000SS, Lode, Groningen, The Netherlands) using a cardiopulmonary exercise testing (CPET) system (Vmaxs-29C, CareFusion 207, Palm Springs, CA, USA) [18]. Incremental testing was performed, which consisted of 2-min increments to 10 W. Subsequent constant-load testing, which was conducted at 70% of the maximal incremental work rate obtained in pre-treatment, was performed to measure the exertional inspiratory capacity (IC) at pre-treatment and post-treatment with the same work rate. CPET was performed under room air conditions until subject exhaustion. Expired gas data were measured breath-by-breath and collected as 30-s averages at rest, during exercise at 2-min intervals, and at end-exercise. In addition, dyspnea and leg fatigue intensity (Borg scale) were evaluated, and arterial blood was collected at rest and during the last 15 s of each exercise stage and at end-exercise. Arterial blood samples for blood gas analyses, plasma norepinephrine, and plasma lactate were obtained and measured as previously described [18]. Before ghrelin administration, blood samples were also collected after 30 min of bed rest in the morning following an overnight fast for measurement of plasma acyl-ghrelin by radioimmunoassay, as previously described [19].

### Dual-Energy X-ray Absorptiometry (DEXA)

Dual energy x-ray absorptiometry (DEXA) was performed to assess total body composition, including lean body mass before and after ghrelin or placebo administration with PR, as previously described [16].

### Respiratory and peripheral muscle strength

The maximal inspiratory pressure (MIP) and maximal expiratory pressure (MEP) were measured, as described previously [16].

### Sample size

The main multicenter study’s target sample size was 60 in the original protocol at the time of study design [16]. Before the main multicenter study ended, the power and sample size calculations were repeated using the estimated effect of only ghrelin treatment for improving 6-min walk distance (6-MWD), which was based on information from the pilot study [15,16]. The recalculated sample size was 18 in the main multicenter trial. What constituted a clinically important change in peak $\overset{•}{V}o_{2}$ after ghrelin treatment with PR was not known before the study ended. Therefore, a formal power calculation could not be done. As an exploratory study, it was confirmed that the number of patients that had completed the study in our center exceeded the number necessary for the re-calculated sample size of 18, though the calculation was not based on the primary outcome in the present study.

### Data analysis

Two measurement points were used for evaluations of exercise parameters: iso-time and peak exercise point. “iso-time” was defined as the highest equivalent exercise time achieved during the CPETs performed at pre-treatment and Week 3 by a given subject. To obtain “iso-time” during exercise, the values of the cardio-respiratory parameters were calculated by linear interpolation between adjacent measurement points for each subject [20,21]. The absolute values of the cardio-respiratory parameters were obtained during CPET at 2-min intervals, and at end-exercise.

### Statistical analysis

All data are expressed as means ± SD unless otherwise indicated. Comparisons of baseline characteristics between the groups were made by Fisher’s exact tests and Wilcoxon rank sum tests. Effects were examined once, at Week 3 soon after 3-week treatment. The results at Week 3 were compared with the pre-treatment results within each group, and between the two groups using paired *t*-tests and unpaired *t*-tests, respectively. To assess the time-course efficacy of ghrelin versus placebo, post-treatment data were also analyzed using repeated-measures analysis of variance (ANOVA). Spearman’s rank correlation coefficients were used to assess the relationships between the increase in the peak $\overset{•}{V}o_{2}$ and other measurements. The statistical analysis of the present study was performed by Mr. K. Tsuguchi (Nihon Ultmarc Inc. Tokyo, Japan) who had no relevant conflicts of interest. A p value <0.05 was considered significant (SAS 9.1.3, SAS Institute Inc., Cary, NC, USA).

## Results

A total of 20 underweight patients (mean BMI (SD), 18.3 (2.3) kg/m^2^) was randomized to ghrelin treatment with PR (ghrelin group; n = 10) or placebo treatment with PR (placebo group; n = 10), and all patients completed the study protocol. There were no significant differences in baseline characteristics between the two groups (Table 1). No significant difference in the total work performed during 3-week PR (mean ± SD: ghrelin group, 6970 ± 6169 watt·min versus placebo group, 7167 ± 4310 watt·min, between-group p = 0.935) was observed. Next, the effects of ghrelin administration on exercise capacity, as well as the underlying mechanisms were examined once (Week 3) using two measurement points: iso-time and the peak exercise point.

**Table 1:** Patients’ baseline characteristics*

	**Ghrelin, n = 10**	**Placebo, n = 10**
Age (years)	70.8 (6.4)	73.1 (5.6)
Sex (males/females)	10/0	9/1
BMI (kg/m^2^)	18.4 (2.4)	18.1 (2.3)
Cigarettes (pack-years)	59.1 (29.2)	64.8 (25.1)
Pulmonary function		
FEV1 (L)	0.83 (0.21)	0.82 (0.23)
%FEV1 (% predicted)	32.0 (9.4)	33.3 (10.5)
FEV1/FVC (%)	41.7 (7.6)	42.4 (8.0)
VC (L)	2.54 (0.42)	2.62 (0.50)
%VC (%)	79.6 (11.0)	86.1 (15.3)
IC (L)	1.63 (0.22)	1.74 (0.38)
DLco (% predicted)	61.3 (23.8)	72.4 (27.4)
Exercise capacity in ICPET		
Peak work rate (Watt)	36.0 (13.5)	34.0 (9.7)
Peak $\overset{•}{V}o_{2}\left(\mathrm{mL}/\mathrm{kg}/\min\right)$	13.3 (3.7)	13.4 (3.2)
Medications		
LAMA	7	5
SAMA	2	2
LABA	6	5
ICS	4	1
Methylxanthines	5	6
Comorbidity		
Angina pectoris	2	0
PVC	0	1

Data are presented as means (SD) unless otherwise stated. *BMI* Body mass index, *DLco* Carbon monoxide diffusing capacity, *FEV*_*1*_ Forced Expiratory Volume in one second, *FVC* Forced Vital Capacity, *IC* Inspiratory Capacity, *ICPET* Incremental Cardiopulmonary Exercise Test, *ICS* Inhaled Corticosteroids, *LABA* Long-Acting β_2_-Agonist, *LAMA* Long-Acting Muscarinic Antagonist, *PVC* Premature Ventricular Contraction, *SAMA* Short-Acting Muscarinic Antagonist, *VC* Vital Capacity. * The groups shown represent all treated patients. Medications were not mutually exclusive, and data are presented separately.

### Peak incremental exercise responses

**Figure 2:**
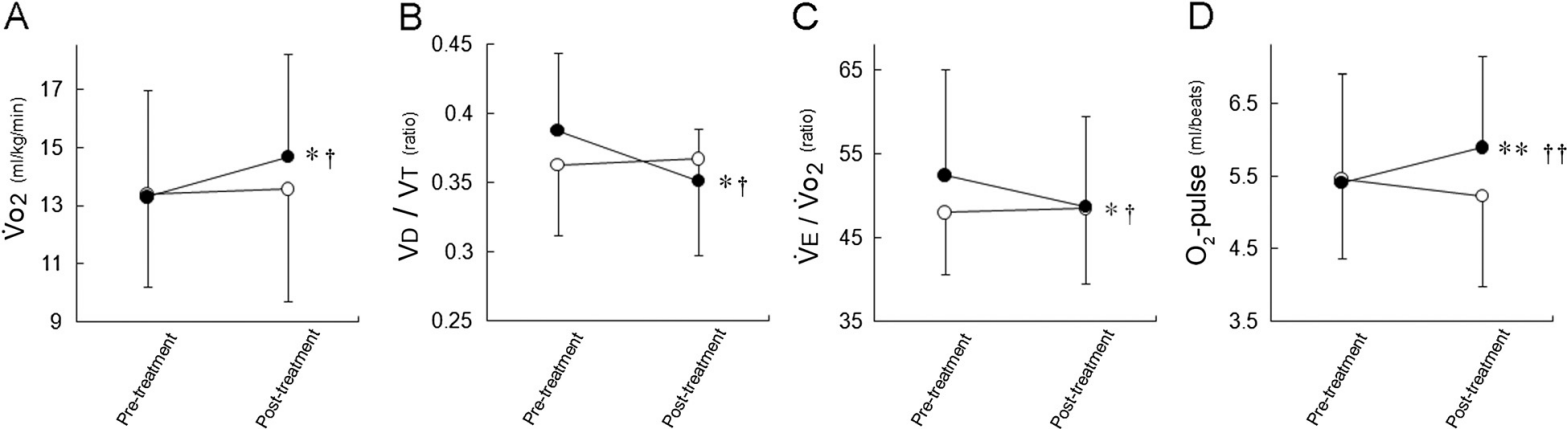
**With incremental exercise, cardiopulmonary parameters at peak exercise before and after 3-week administration of ghrelin or placebo with PR.** Solid symbols, ghrelin; Open symbols, placebo. Data are presented as means ± SD. * p < 0.05, ** p < 0.01: change between pre-treatment and post-treatment (between ghrelin and placebo group difference, treatment effect) by unpaired *t*-test: ^†^ p < 0.05, ^††^ p < 0.01: repeated-measures ANOVA indicates significant time-course effects of ghrelin versus placebo. $\overset{•}{V}o_{2}$, oxygen uptake; V_D_/V_T_, physiologic dead space/tidal volume ratio, $\overset{•}{V}E$, minute ventilation; V_T_, tidal volume.

The distribution of reasons for stopping exercise was not different after ghrelin than after placebo; most subjects in both groups stopped primarily because of dyspnea or a combination of dyspnea and leg discomfort (ghrelin group, 80% versus placebo group, 60%), and fewer subjects stopped because of leg discomfort (ghrelin group, 20% versus placebo group, 40%). The ghrelin-PR combination substantially increased the peak $\overset{•}{V}o_{2}$ (mean difference (ghrelin minus placebo), i.e., treatment effect = 1.2 mL/kg/min, 95% CI: 0.2 to 2.3 mL/kg/min, between-group p = 0.025, Table 2 and Figure 2A, or as an alternative description, treatment effect = 60.5 mL/kg, 95% CI: 10.5 to 110.5 mL/kg, between-group p = 0.020). Additionally, repeated-measures analysis of variance (ANOVA) indicated a significant time-course effect of ghrelin versus placebo in the peak $\overset{•}{V}o_{2}$(p = 0.025). After ghrelin-PR combination treatment, the peak $\overset{•}{V}o_{2}$ increased by >2 mL/kg/min in two patients and decreased in none. Furthermore, compared with placebo, the ghrelin-PR combination significantly increased $\overset{•}{V}o_{2}/\mathrm{HR}$ (treatment effect = 0.7 mL/beat, 95% CI: 0.3 to 1.2 mL/beat, between-group p = 0.003) and decreased V_D_/V_T_ (treatment effect = -0.04, 95% CI: -0.08 to -0.00, between-group: p = 0.041), $\overset{•}{V}E\overset{•}{/V}co_{2}$ (treatment effect = -4.1, 95% CI: -8.2 to -0.1, between-group: p = 0.045), and $\overset{•}{V}E/\overset{•}{V}o_{2}$ (treatment effect = -4.2, 95% CI: -7.9 to -0.5, between-group: p = 0.030), but maintained the dyspnea rating unchanged (Table 2 and Figure 2B-2D).

**Table 2:** Changes in peak incremental exercise parameters after pulmonary rehabilitation with ghrelin or placebo

	**Ghrelin, n = 10**		**Placebo, n = 10**		**Treatment effect (95% CI; p value**^**†**^**)**
	**Pre-treatment**	**Mean D.**	**Pre-treatment**	**Mean D.**	
$\overset{•}{V}o_{2}\;\left(\mathrm{mL}/\mathrm{kg}/\min\right)$	13.3 (3.7)	1.4 (0.8)^***^	13.4 (3.2)	0.2 (1.3)	1.2 (0.2 to 2.3; 0.025)
$\overset{•}{V}o_{2}\;\left(\mathrm{mL}/\min\right)$	640.3 (212.5)	69.4 (45.9)	616.8 (138.8)	8.9 (59.7)	60.5 (10.5 to 110.5; 0.020)
Endurance time (s)	389 (157)	48 (96)	372 (131)	59 (60)^*^	-12 (-87 to 63; 0.749)
Dyspnea (Borg)	7.4 (1.5)	-0.5 (1.3)	7.2 (1.6)	-0.9 (1.8)	0.4 (-1.1 to 1.9; 0.572)
Leg discomfort (Borg)	5.5 (2.7)	-0.8 (1.8)	6.3 (2.5)	-0.6 (1.7)	-0.2 (-1.9 to 1.5; 0.803)
V_T_ (L)	0.97 (0.17)	0.10 (0.08)^**^	1.05 (0.12)	0.07 (0.23)	0.03 (-0.13 to 0.19; 0.694)
f (breaths/min)	34 (7)	-2 (3)	28 (5)	-1 (6)	-1 (-5 to 4; 0.802)
$\overset{•}{V}E\;\left(L/\min\right)$	31.9 (6.0)	1.5 (2.7)	29.2 (6.4)	0.5 (2.2)	1.0 (-1.3 to 3.3; 0.389)
V_D_/V_T_	0.39 (0.06)	-0.04 (0.04)^*^	0.36 (0.05)	0.00 (0.04)	-0.04 (-0.08 to -0.00; 0.041)
$\overset{•}{V}E/\overset{•}{V}o_{2}$	52.5 (12.7)	-3.7 (3.7)^*^	48.0 (7.2)	0.5 (4.2)	-4.2 (-7.9 to -0.5; 0.030)
$\overset{•}{V}E\overset{•}{/V}co_{2}$	51.0 (13.6)	-3.8 (3.9)^*^	45.3 (7.5)	0.4 (4.6)	-4.1 (-8.2 to -0.1; 0.045)
Pao_2_ (mmHg)	64.4 (16.0)	-0.5 (4.2)	66.1 (11.9)	-2.2 (5.4)	1.7 (-2.9 to 6.2; 0.454)
Paco_2_ (mmHg)	42.7 (7.0)	-0.3 (2.3)	42.4 (4.2)	0.7 (3.0)	-0.9 (-3.4 to 1.6; 0.453)
HR (beats/min)	118 (14)	2 (8)	113 (13)	7 (7)^*^	-6 (-13 to 2; 0.115)
$\overset{•}{V}o_{2}/\mathrm{HR}\left(\mathrm{mL}/\mathrm{beats}\right)$	5.4 (1.5)	0.5 (0.5)^*^	5.5 (1.1)	-0.2 (0.5)	0.7 (0.3 to 1.2; 0.003)
Plasma LT (mg/dL)	32.3 (11.8)	3.3 (7.6)	35.0 (14.5)	2.5 (9.8)	0.9 (-7.4 to 9.1; 0.824)
Plasma NE (ng/mL)	2.45 (1.70)	-0.08 (1.29)	2.65 (1.80)	-0.11 (0.84)	0.03 (-0.99 to 1.06; 0.945)

Data are presented as means (SD) unless otherwise indicated. *D* Difference from pre-treatment, *f* breathing frequency, H*R* Heart Rate, *LT* Plasma Lactate level, *NE* Plasma norepinephrine level, *Paco*_*2*_ Arterial carbon dioxide tension, *Pao*_*2*_ Arterial oxygen tension, Treatment effect, Mean difference (ghrelin minus placebo), *V*_*D*_*/V*_*T*_ physiologic dead space/tidal volume ratio, $\overset{•}{V}E$ Minute ventilation, $\overset{•}{V}o_{2}$ oxygen uptake, *V*_*T*_ Tidal volume. * p < 0.05, ** p < 0.01, *** p < 0.001: change between pre-treatment and post-treatment (within-group difference) by paired *t*-test. ^†^ change between pre-treatment and post-treatment (treatment effect) by unpaired *t*-test.

### Iso-time responses with incremental exercise

The ghrelin-PR combination significantly decreased the dyspnea score from baseline, but not compared to placebo (Table 3). The ghrelin-PR combination decreased V_D_/V_T_ compared to placebo (treatment effect = -0.04, 95% CI: -0.08 to -0.00, between-group p = 0.048) and $\overset{•}{V}E/\overset{•}{V}o_{2}$ (within group: p < 0.05), and increased V_T_ (within-group: p < 0.01), with a resultant decrease in the respiratory frequency (f) (within-group p < 0.05) (Table 3). The ghrelin-PR combination did not inhibit plasma norepinephrine levels.

**Table 3:** Changes in iso-time parameters during incremental exercise after pulmonary rehabilitation with ghrelin or placebo

	**Ghrelin, n = 10**		**Placebo, n = 10**		**Treatment effect (95% CI; p value**^**†**^**)**
	**Pre-treatment**	**Mean D.**	**Pre-treatment**	**Mean D.**	
$\overset{•}{V}o_{2}\left(\mathrm{mL}/\mathrm{kg}/\min\right)$	13.2 (3.7)	0.5 (1.8)	13.4 (3.2)	-0.8 (1.1)	1.2 (-0.2 to 2.6; 0.085)
Dyspnea (Borg)	7.2 (1.5)	-2.0 (2.6)^*^	7.0 (1.7)	-2.7 (3.2)^*^	0.7 (-2.1 to 3.4; 0.606)
Leg discomfort (Borg)	5.4 (2.7)	-1.9 (2.9)	6.2 (2.6)	-3.0 (2.9)^*^	1.1 (-1.6 to 3.9; 0.399)
V_T_ (L)	0.97 (0.16)	0.10 (0.08)^**^	1.06 (0.12)	0.09 (0.24)	0.01 (-0.15 to 0.18; 0.876)
f (breaths/min)	34 (7)	-4 (6)^*^	28 (5)	-3 (8)	-1 (-8 to 5; 0.658)
$\overset{•}{V}E\left(L/\min\right)$	31.8 (6.1)	-0.4 (4.6)	29.2 (6.5)	-1.6 (3.6)	1.2 (-2.6 to 5.1; 0.510)
V_D_/V_T_	0.39 (0.06)	-0.03 (0.04)^*^	0.36 (0.05)	0.01 (0.04)	-0.04 (-0.08 to -0.00; 0.048)
$\overset{•}{V}E/\overset{•}{V}o_{2}$	52.5 (12.7)	-3.3 (3.6)^*^	47.9 (7.2)	0.2 (4.9)	-3.5 (-7.6 to 0.5; 0.086)
$\overset{•}{V}E\overset{•}{/V}co_{2}$	51.1 (13.7)	-2.7 (5.3)	45.2 (7.5)	0.9 (5.2)	-3.6 (-8.6 to 1.4; 0.149)
Pao_2_ (mmHg)	64.5 (16.0)	1.0 (5.4)	66.2 (11.8)	-1.1 (4.8)	2.1 (-2.7 to 7.0; 0.361)
Paco_2_ (mmHg)	42.6 (6.9)	-1.1 (2.4)	42.4 (4.2)	0.0 (3.1)	-1.2 (-3.8 to 1.4; 0.359)
HR (beats/min)	118 (14)	-2 (9)	113 (13)	2 (6)	-4 (-11 to 4; 0.290)
$\overset{•}{V}o_{2}/\mathrm{HR}\left(\mathrm{mL}/\mathrm{beats}\right)$	5.4 (1.5)	0.2 (0.8)	5.5 (1.1)	-0.3 (0.4)^*^	0.5 (-0.1 to 1.1; 0.093)
Plasma LT (mg/dL)	32.1 (11.8)	-0.3 (8.3)	34.9 (14.6)	-3.5 (7.9)	3.2 (-4.4 to 10.8; 0.920)
Plasma NE (ng/mL)	2.44 (1.71)	-0.37 (1.40)	2.64 (1.81)	-0.77 (1.06)^*^	0.40 (-0.77 to 1.56; 0.426)

Data are presented as means (SD) unless otherwise indicated. *D*, Difference from pre-treatment; *f*, breathing frequency; *HR*, Heart Rate; *LT*, Plasma Lactate level; *NE*, Plasma Norepinephrine level; *Paco2*, Arterial carbon dioxide tension; *Pao2*, Arterial oxygen tension; *Treatment effect*, Mean difference (ghrelin minus placebo); *Vd/Vt*, Physiologic dead space/tidal volume ratio; $\overset{•}{V}E$ Minute ventilation, $\overset{•}{V}o_{2}$ Oxygen uptake; *VT*, Tidal volume. * p < 0.05, ** p < 0.01: change between pre-treatment and post-treatment (within-group difference) by paired *t*-test. ^†^ change between pre-treatment and post-treatment (treatment effect) by unpaired *t*-test.

### The responses on dynamic hyperinflation with constant load exercise

The ghrelin-PR combination significantly increased IC at the iso-time point (within-group p < 0.05) and peak exercise (within-group p < 0.05), though the treatment effect compared with placebo was not significant.

### Correlates of improvement

The intra-subject increase in the peak $\overset{•}{V}o_{2}$ after ghrelin treatment correlated with the plasma acyl-ghrelin level after treatment (r = -0.797, p = 0.006), which in turn correlated with the increase induced by treatment in the plasma acyl-ghrelin level (r = -0.684, p = 0.029) and minute ventilation $\left(\overset{•}{V}E\right)$ (r = 0.657, p = 0.039).

## Discussion

This substudy of a multicenter, randomized, double-blind, placebo-controlled trial of ghrelin treatment in underweight COPD patients was designed and powered to investigate the effects of repeated ghrelin administration and the underlying mechanisms using cardiopulmonary exercise testing. The main findings were as follows. 1) With incremental exercise, in the ghrelin group, the mean change from pre-treatment i) in peak $\overset{•}{V}o_{2}$ was significantly increased compared with the placebo group, and ii) that in dyspnea intensity showed a significant within-group decrease at the iso-time point. Furthermore, iii) the mean changes from pre-treatment in $\overset{•}{V}E\overset{•}{/V}co_{2}$, $\overset{•}{V}E/\overset{•}{V}o_{2}$, and V_D_/V_T_ were significantly improved compared with the placebo group at peak exercise; iv) the mean change from pre-treatment in V_T_ showed a significant within-group increase at peak exercise; and v) that in O_2_-pulse was increased compared with the placebo group at peak exercise. 2) With constant-load exercise, in the ghrelin group, the mean change from pre-treatment in IC showed a significant within-group increase throughout exercise.

Many approaches to treating cachectic COPD have been attempted [2,7]. In the present study, exercise training alone did not show significant improvements in exertional capacity and cardio-ventilatory parameters, except for endurance time. In the placebo group, $\overset{•}{V}o_{2}$ (mean difference: -0.8 mL/kg/min, within-group: p = 0.051) and O_2_-pulse were not improved at the iso-time point (Table 3). The plasma norepinephrine levels seemed to be inhibited at the iso-time point (Table 3). Given the reported negative connotations of sympathetic activation in cardiac as well as respiratory disease patients [22-26], sympathetic inhibition may have positive consequences in COPD. These findings suggest that underweight patients with COPD who undergo exercise training can only increase their endurance time, efficiently decreasing $\overset{•}{V}o_{2}$ and plasma norepinephrine levels. In contrast, repeated ghrelin administration was associated with a significant increase from pre-treatment in the peak $\overset{•}{V}o_{2}$ by an average of 1.4 mL/kg/min (between-group p = 0.025, Table 2 and Figure 2A). Endurance time was not increased after ghrelin-PR combination treatment (Table 2). These findings suggest that patients treated with ghrelin had a high exercise capacity with instantaneous force, though the pedal revolutions per minute on the electrically braked-cycle ergometer were not monitored. We considered the following potential mechanisms for the increase in exercise capacity that was induced by ghrelin treatment: i) improved ventilatory efficiency and ventilatory volume; and ii) increased cardiac function.

The two major contributing factors leading to exercise limitation in COPD may be the increased ventilatory requirement and the decreased ventilatory capacity [27]. In the ghrelin group, V_D_/V_T_, $\overset{•}{V}E\overset{•}{/V}co_{2}$, and $\overset{•}{V}E/\overset{•}{V}o_{2}$, which are useful to estimate wasted ventilation, were significantly decreased at peak exercise (Table 2, Figure 2B and C), resulting in a decreased ventilatory requirement. These findings suggest that ghrelin treatment was associated with an improvement of ventilatory efficiency at peak exercise. V_T_ during incremental exercise (Tables 2 and 3) and IC during constant-load exercise were increased after ghrelin-PR combination treatment, though the difference compared with placebo was not significant. Additionally, increased peak $\overset{•}{V}o_{2}$ after ghrelin treatment was positively correlated with the change in $\overset{•}{V}E$ from pre-treatment. In the present study, 4 weeks after the completion of ghrelin and PR treatment, MEP was better in the ghrelin group than in the placebo group (Table 4). These findings suggest that these improvements in ventilatory parameters may occur through the mechanism by which ghrelin improved respiratory muscle strength [16]. On the other hand, ghrelin may have additional beneficial effects within the cardiovascular system in underweight COPD patients. Earlier studies showed that ghrelin improved cardiac function in normal subjects and in patients/animals with heart failure [13,28,29]. In the ghrelin group, the O_2_-pulse after treatment increased significantly compared with placebo at peak incremental exercise. Furthermore, a recent study demonstrated that ghrelin acts on the central nervous system to attenuate sympathetic nervous activity [14], though in the present study, as well as in the main study, ghrelin treatment did not show significant sympathetic inhibition compared with placebo. Further studies are needed to confirm the clinical significance such as regulating exertional dyspnea. Nevertheless, we believe that these improvements in cardio-ventilatory parameters could represent the effect of ghrelin.

**Table 4:** Changes in pre-treatment resting parameters during pulmonary rehabilitation with ghrelin or placebo

	**Ghrelin, n = 10**		**Placebo, n = 10**		**Treatment effect (95% CI; p value**^**†**^**)**
	**Pre-treatment**	**Mean D.**	**Pre-treatment**	**Mean D.**	
Body weight (kg)	48.2 (7.4)	-0.1 (1.0)	46.8 (7.6)	0.1 (1.3)	-0.2 (-1.3 to 0.9; 0.698)
Total lean mass (kg)	37.2 (5.6)	0.1 (2.3)	35.5 (4.7)	0.6 (0.8)	-0.5 (-2.1 to 1.2; 0.561)
MEP^‡^ (cmH_2_0)	77.7 (28.7)	16.2 (15.8)	87.2 (25.6)	-6.6 (13.4)	22.8 (7.1 to 38.4; 0.007)
MIP^‡^ (cmH_2_0)	-52.7 (20.8)	-10.0 (24.6)	-54.2 (15.4)	-7.9 (5.4)	-2.1 (-22.5 to 18.2; 0.825)
PFT					
FEV1 (L)	0.83 (0.21)	0.06 (0.16)	0.82 (0.23)	0.07 (0.16)	-0.01 (-0.16 to 0.13; 0.845)
FEV1/FVC (%)	41.7 (7.6)	-1.9 (3.6)	42.4 (8.0)	-3.4 (3.8)^*^	1.5 (-2.1 to 5.0; 0.383)
%FEV1 (%)	32.0 (9.4)	2.1 (6.3)	33.3 (10.5)	2.9 (6.3)	-0.8 (-6.8 to 5.1; 0.778)
VC (L)	2.54 (0.42)	0.18 (0.28)	2.62 (0.50)	0.15 (0.20)^*^	0.03 (-0.20 to 0.26; 0.800)
%VC (%)	79.6 (11.0)	5.5 (8.9)	86.1 (15.3)	4.9 (7.3)	0.6 (-7.1 to 8.2; 0.877)
DLco (%)	61.3 (23.8)	10.0 (37.5)	72.4 (27.4)	-6.1 (12.2)	16.1 (-10.1 to 42.3; 0.222)

Data are presented as means (SD) unless otherwise indicated. *D* Difference from pre-treatment, *DLco* Carbon monoxide diffusing capacity, *FEV*_*1*_ Forced Expiratory Volume in one second, *FVC* Forced Vital Capacity, *MEP* Maximal Expiratory Pressure, *MIP* Maximal Inspiratory Pressure, *PFT* Pulmonary Function Test, *Treatment effect* Mean Difference (ghrelin minus placebo), *VC* Vital Capacity. * p < 0.05, *** p < 0.001: change between pre-treatment and post-treatment (within-group difference) by paired *t*-test. ^†^ change between pre-treatment and post-treatment (treatment effect) by unpaired *t*-test. ^‡^ MEP and MIP were tested at pre-treatment and 4 weeks after the completion of the combination treatment.

It should be noted that individuals in this study had different reactions to PR or ghrelin therapy. Of note, in the present study, the exercise capacity improvement by exogenous ghrelin was negatively correlated with the increase in the plasma acyl-ghrelin level after treatment (r = -0.684). Since the half-life of exogenous ghrelin, which was administered on the day before blood sampling, was about 20 min [30], it is highly possible that the measured plasma acyl-ghrelin level was unaffected by the administered exogenous ghrelin, and reflected the endogenous acyl-ghrelin. These correlations may stand to reason, given that ghrelin administration is considered as replacement therapy for underweight COPD patients who may be unable to compensate for the anabolic-catabolic imbalance, resulting in increasing the endogenous plasma acyl-ghrelin levels, thought repeated exogenous ghrelin might inhibit ghrelin production [31]. Meanwhile, as an effective adjunctive therapy, we used exercise training as a part of PR, which is well accepted to improve exercise performance [8,32]. However, it is important to determine whether PR improves or worsens it, especially in underweight patients in the severe disease stage.

This study had some limitations. First, the number of participants was small, and few women were included in this trial. Given the exploratory nature of the present study, a formal power calculation was not possible. Many statistical comparisons under exercise conditions might increase the chance of type I error. As a consequence, the present findings should be considered preliminary and need to be confirmed in a future large study. Second, ghrelin did not show any effects on whole lean body mass and weight. The training work rate remained at the initial setting in 4 patients (40%) in each group during this trial, because they found the initial training work rate intolerable. The lack of response to exercise training or ghrelin treatment in weight and whole lean body mass may be related to the inability to tolerate the type, the intensity, and/or the duration of the exercise training. Third, there was a significant difference between the ghrelin and placebo groups in the peak $\overset{•}{V}o_{2}$ measured by the protocol of 2-min increments to 10 W in the present study. However, in the main multicenter trial at Week 3, outcome measurements measured with a continuous ramp rate of 5 W/min showed no improvements with ghrelin compared to placebo [16]. In the present study, in the ghrelin group, the mean change from pre-treatment in the peak $\overset{•}{V}o_{2}$ measured by the protocol of 2-min increments to 10 W was larger than that measured by the protocol with the ramp rate of 5 W/min (mean difference: by 2-min increments to 10 W, 1.4 mL/kg/min versus with a ramp rate of 5 W/min, 1.1 mL/kg/min). See Table E1 in Additional file 2 (Supplementary Results) for other details. Several investigators have suggested the desirability of adjusting the work rate increment in exercise testing according to the patient’s cardiopulmonary condition [33,34]. Tests in which the work rate increased too rapidly may not allow sufficient data for the evaluation to be accumulated. A more suitable exercise testing protocol, considering the patient’s cardiopulmonary condition, should have been conducted in the main multicenter trial. Also, in the present study, at Week 7, i.e., 4 weeks after the completion of the treatment, CPET should have been conducted to evaluate the sustained effects of ghrelin treatment, as shown in the multicenter trial [16].

## Conclusion

Given the short-term nature of the study and the discrepancy between the physiological findings and the measures of direct patients benefit, the present findings need to be confirmed by further study. However, in the present substudy, ghrelin administration was associated with improved exertional capacity and improvements in cardio-ventilatory parameters, although the main multicenter study showed no significant difference between the ghrelin and placebo group in exercise capacity. Development of ghrelin administration as a novel cachexia-targeted therapy capable of improving exercise performance may be helpful in COPD treatment.

## Abbreviations

BMI: Body mass index; BP: Blood pressure; COPD: Chronic obstructive pulmonary disease; CPET: Cardiopulmonary exercise testing; D: Difference; DLco: Carbon monoxide diffusing capacity; F: Breathing frequency; FEV1: Forced expiratory volume in one second; FVC: Forced vital capacity; GH: Growth hormone; HR: Heart rate; IC: Inspiratory capacity; ICS: Inhaled corticosteroids; LABA: Long-acting β_2_-agonist; LAMA: long-acting muscarinic antagonist; LT: plasma lactate level; MVV: Maximum voluntary ventilation; NE: Plasma norepinephrine level; $\overset{•}{V}o_{2}/\mathrm{HR}$: O_2_-pulse; Paco2: Arterial carbon dioxide tension; Pao2: Arterial oxygen tension; PFT: Pulmonary function test; PR: Pulmonary rehabilitation; SABA: Short-acting β_2_-agonist; SAMA: Short-acting muscarinic antagonist; VC: Vital capacity; VD/VT: Physiologic dead space/tidal volume ratio; $\overset{•}{V}E$: Minute ventilation; $\overset{•}{V}E\overset{•}{/V}co_{2}$: Ventilatory equivalent for carbon dioxide; $\overset{•}{V}E/\overset{•}{V}o_{2}$: Ventilatory equivalent for oxygen; $\overset{•}{V}o_{2}$: Oxygen uptake; VT: Tidal volume

## Competing interests

The authors declare that they have no competing interests.

## Authors’ contributions

KM, RM, NN, SK, M. Miki, KY, YT, M. Motone, TH, M. Mori, and KK conceived and designed the experiments. KM, RM, SK, M. Miki, KY, YT, M. Motone, TH, M. and Mori performed the experiments. KM, RM, NN, SK, M. Miki, KY, YT, M. Motone, TH, M. Mori, and KK analyzed the data. KM wrote the manuscript. All authors read and approved the final manuscript.

## Pre-publication history

The pre-publication history for this paper can be accessed here:

http://www.biomedcentral.com/1471-2466/13/37/prepub

## Supplementary Material

Additional file 1 Effects of Ghrelin Treatment on Exercise Capacity in Underweight COPD Patients: a substudy of a multicenter, randomized, double-blind, placebo-controlled trial of ghrelin treatment. Click here for file

Additional file 2 Effects of Ghrelin Treatment on Exercise Capacity in Underweight COPD Patients: a substudy of a multicenter, randomized, double-blind, placebo-controlled trial of ghrelin treatment. Click here for file
