# Supplementary material for: Effects of Ghrelin Treatment on Exercise Capacity in Underweight COPD Patients: a substudy of a multicenter, randomized, double-blind, placebo-controlled trial of ghrelin treatment
Source: BMC Pulm Med. 2013 Jun 10;13:37. doi: 10.1186/1471-2466-13-37 (PMC3683541; doi:10.1186/1471-2466-13-37)
Supplement: Additional file 1 — Effects of Ghrelin Treatment on Exercise Capacity in Underweight COPD Patients: a substudy of a multicenter, randomized, double-blind, placebo-controlled trial of ghrelin treatment. [file 1471-2466-13-37-S1.doc]

**Physiological** **Effects of Ghrelin in Cachectic COPD: substudy of a multicenter, randomized, double-blind, placebo-controlled trial of ghrelin treatment**

Keisuke Miki, Ryoji Maekura, Noritoshi Nagaya, Seigo Kitada, Mari Miki, Kenji Yoshimura, Yoshitaka Tateishi, Masaharu Motone, Toru Hiraga, Masahide Mori, Kenji Kangawa.

**Supplementary Protocol**

**Clinical Trial of Ghrelin Administration in Patients with**

**Chronic Obstructive Pulmonary Disease:**

Date of final protocol: July 1, 2005 Keisuke Miki

　　　　　　　　　　　　　　　　　　　　　　　　　　　　 Ryoji Maekura

**-Contents-**

Page

Schema 4

Summary of the clinical trial 5

I Background 8

II Objectives of the clinical trial 13

III Clinical trial team (multicenter trial) 13

IV The clinical trial 18

V The clinical trial program 21

VI Adverse Events 26

VII Ethical conduct of the study 28

VIII Discontinuation, suspension, or completion of the clinical trial 31

IX Statistics Analyses 31

X Audit of the clinical trial 32

XI Payment and compensation for health damage 32

XII Intellectual property 32

XIII Items related to the Act on the Protection of Personal Information Held by

Administrative Organs 32

Contact information (secretariat) 33

**Appendix 1**: The information sheet of “Clinical Trial of Ghrelin Administration in patients

with Chronic Obstructive Pulmonary Disease” 34

**Appendix 2**: The informed consent form of “Clinical Trial of Ghrelin Administration in

patients with Chronic Obstructive Pulmonary Disease ” 38

**Appendix 3:** Request form for handling (discard/change) samples of “Clinical Trial of

Ghrelin Administration in Patients with Chronic Obstructive Pulmonary Disease” 39

**Schema**

**Enrollment** (n = 20)

**Randomized allocation**

**Placebo group** (n = 10) **Ghrelin group** (n = 10)

3-week treatment 3-week treatment

Pulmonary rehabilitation Pulmonary rehabilitation

+ +

Sterile saline 10ml Human ghrelin 2μg/kg

twice a day ghrelin solution

with 10 ml Sterile saline 　　　　　　　twice a day

**Evaluation**

Measurements soon after 3-week treatment

**Summary of the clinical trial**

**1) Title of the clinical trial**

Clinical trial of ghrelin administration in patients with chronic obstructive pulmonary disease: substudy of a multicenter, randomized, double-blind, placebo-controlled trial

**2) Objectives of the clinical trial**

The efficacy of adding synthetic human ghrelin or sterile saline to pulmonary rehabilitation (PR) for 3 weeks in cachectic chronic obstructive pulmonary disease (COPD) patients on exercise capacity and the underlying mechanisms were investigated. (This study was simultaneously conducted in a single center as part of a multicenter, randomized, double-blind, placebo-controlled study of cachectic COPD patients.)

**3) Trial Inclusion Criteria**

(a) Severe to very severe COPD (forced expiratory volume in one second (FEV1)/forced vital capacity (FVC) of less than 70% and FEV1 percent predicted of less than 50%)

(b) Underweight (body mass index (BMI) < 21 kg/m^2^)

(c) Clinically stable and able to participate in pulmonary rehabilitation

(d) Between 20 and 85 years old

(e) Signed the agreement for participation in this study

**4) Trial Exclusion Criteria**

**:** Participants were excluded for any of the following.

1. Malignant tumours
2. Active infection
3. Severe heart disease
4. Hepatic dysfunction (serum aspartate aminotransferase and alanine

aminotransferase levels at least twice the upper limit of normal)

1. Renal dysfunction (serum creatinine levels ≥ 2.0 mg/dl)
2. Asthma
3. Definitely or possibly pregnant
4. Change in drug regimen within 4 weeks before participation in this study
5. Judged to be unable to participate in this study by their physician.

**5) Study drug**

Active drug: Synthetic human ghrelin (2μg/kg) + sterile saline

Placebo: Sterile saline

**6) Method of the clinical trial**

(a) Selection of patients

(b) Informed consent

(c) Enrollment

(d) Randomized allocation

(e) Pulmonary rehabilitation+clinical trial medication

(f) Evaluation of the safety and the efficacy

(g) Data analyses

A) **Pulmonary rehabilitation** (on an inpatient basis)

i) Physical therapy

ii) Exercise training

B) Method of clinical trial medication

Active drug or placebo will be administered to subjects.

C) **Schedule of** clinical trial medication

In the ghrelin group, synthetic human ghrelin (2ug/kg, dissolved in 10 ml of sterile saline) is administered intravenously for 30 minutes. The infusion is repeated twice a day (before breakfast and before dinner) for three weeks. In the placebo group, sterile saline (10mL) is administered similarly.

**7) Observation/test items**

(a) Cardiopulmonary exercise testing (CPET)

(b) Pulmonary function test

**8) Outcome Measure**

(a) **Efficacy:**

Primary outcomes: Peak oxygen uptake (Vo_2_) measured by CPET

Secondary outcomes: exertional dyspnea

Plasma norepinephrine levels during exercise

O_2_-pulse (Combino_2_/HR)

Physiologic dead space /tidal volume ratio (VD/VT)

Ventilatory equivalent for oxygen (CombinE/Combino_2_)

Ventilatory equivalent for carbon dioxide (CombinE/Combinco_2_)

Inspiratory capacity (IC)

**9) Target sample size**

Active drug group, 10cases; placebo group, 10 cases; Total cases, 20 cases.

**10) Expected period of the Clinical trial**

From the ethical committee approval in 2005 to June 2008.

**I Background**

**1) Introduction**

Ghrelin was first discovered as a novel growth hormone (GH)-releasing peptide isolated from the stomach in 1999 by Kojima, Kangawa et al. In the subsequent study, it has been reported that expression of ghrelin is also detectable in the plasma, and growth hormone-secretagogue receptor expression is demonstrated in several tissues as well as the hypothalamus and pituitary. It has been reported that, as the physiological functions, ghrelin have 1) GH-releasing activity; 2) sympathoinhibitory action; 3) vasodilatory effects and the increasing effects in cardiac output; 4) orexigenic effect; and 5) regulation effect of energy metabolism. Thus, it has been demonstrated that ghrelin is broadly involved in energy metabolic system. Pulmonary cachexia is common in the advanced stage of chronic obstructive pulmonary disease (COPD), and it is an independent risk factor for death in such patients. These physiological functions raise the possibility that ghrelin administration may also improve pulmonary cachexia in COPD.

**2) Synthetic human ghrelin**

Generic name; synthetic human ghrelin

Molecular formula

O=C-(CH2)6-CH3



O



GSSFLSPEHQRVQQRKESKKPPAKLQPR

Molecular weight; 3,343

Ghrelin consists of 28 amino acids and contains a unique fatty acid modification, n-octanoylation, at Ser 3. (Two residues are different between the human and rat amino acid sequences.)


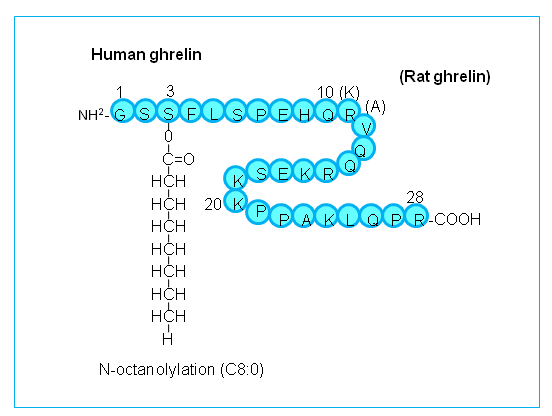


**3) Nonclinical studies**

(a) Pharmacology (stimulation of GH secretion and action on other neuroendocrine secretion)

In rat cultured pituitary cells, synthetic rat ghrelin (Arg^11^-Val^12^ in human ghrelin are substituted for LyS^11^-Ala^12^) exhibited a GH secretion-stimulatory effect (EC_50_ = 2.1nM) equivalent that of GHRH. Ghrelin did not affect secretion of ACTH, FSH, LH, PRL, or TSH at a level up to 10 μM. In male rats, synthetic rat ghrelin (10μg iv) elevated the GH level to a peak of about 140 ng/mL within 5-10 minutes. In this in vivo system, synthetic rat ghrelin did not affect secretion of the other anterior pituitary hormones.

(b) Toxicity

In a toxicity study of ghrelin performed between March 21, 2000 and October 2, 2000 by Daiichi Suntory Pharma Co., Ltd., rat ghrelin was intravenously administered to Crj:CD (SD) IGS rats at 10 and 50 μg/kg (5 animals per group) for 2 weeks, and observation of the general condition, measurement of the body weight, food intake, and water intake, urinalysis, blood test, blood chemistry, bone marrow test, pathological examination, and DNA synthesis test employing BrdU incorporation were performed to investigate the in vivo biochemical action of rat ghrelin, and no influence of rat ghrelin was noted in any of these items. Under the condition of this study, 2-week repeated intravenous administration of rat ghrelin at 10 and 50 μg/kg showed no apparent toxicity.

**4) Clinical trial**

(a) **Pharmacology** (stimulation of GH secretion and action on other neuroendocrine secretion)

In the clinical study involving healthy volunteers performed between January 17, 2001 and April 11, 2001 at National Cerebral and Cardiovascular Center, 10 μg/kg of synthetic human ghrelin elevated the serum GH level to about 80 ng/mL within 30 minutes and then decreased to about 20 ng/mL at 120 minutes. The peak blood ACTH and PRL levels were 215 pg/mL and 20.4 ng/mL, respectively. An Italian group reported that the GH level reached a peak (92.1 ng/mL) 30 minutes after synthetic human ghrelin administration at 1.0 μg/kg.

(b) **Pharmacology** (effect on hemodynamics)

In the clinical study involving 6 healthy volunteers performed at the National Cerebral and Cardiovascular Center, 10 μg/kg synthetic human ghrelin reduced the mean arterial blood pressure by 12 mmHg, showing a significant difference on comparison with the placebo group, but no significant increase was noted in the heart rate.

(c) **Pharmacokinetics**

In the clinical study involving 6 healthy volunteers performed at the National Cerebral and Cardiovascular Center, the blood ghrelin level reached an about 61 times higher level (150 ng/ml) than the baseline one minute after synthetic human ghrelin administration at 10 μg/kg. Administered ghrelin was rapidly eliminated from the circulation with a half-life of about 10 minutes, and the level decreased to mostly the baseline at 120 minutes.

(d) **Toxicity**

In previous clinical studies, intravenous ghrelin administration induced only subjective mild heat sensation and enhanced intestinal peristalsis, and no other particular subjective or objective symptoms were observed. A repeated dose study involving heart disease patients (7 patients as of January 20, 2003) has been performed at National Cerebral and Cardiovascular Center, and no particular adverse event has occurred.

**Physiological functions of ghrelin**

1. **Stimulation of growth hormone release** (Ref. ^E1-3^)


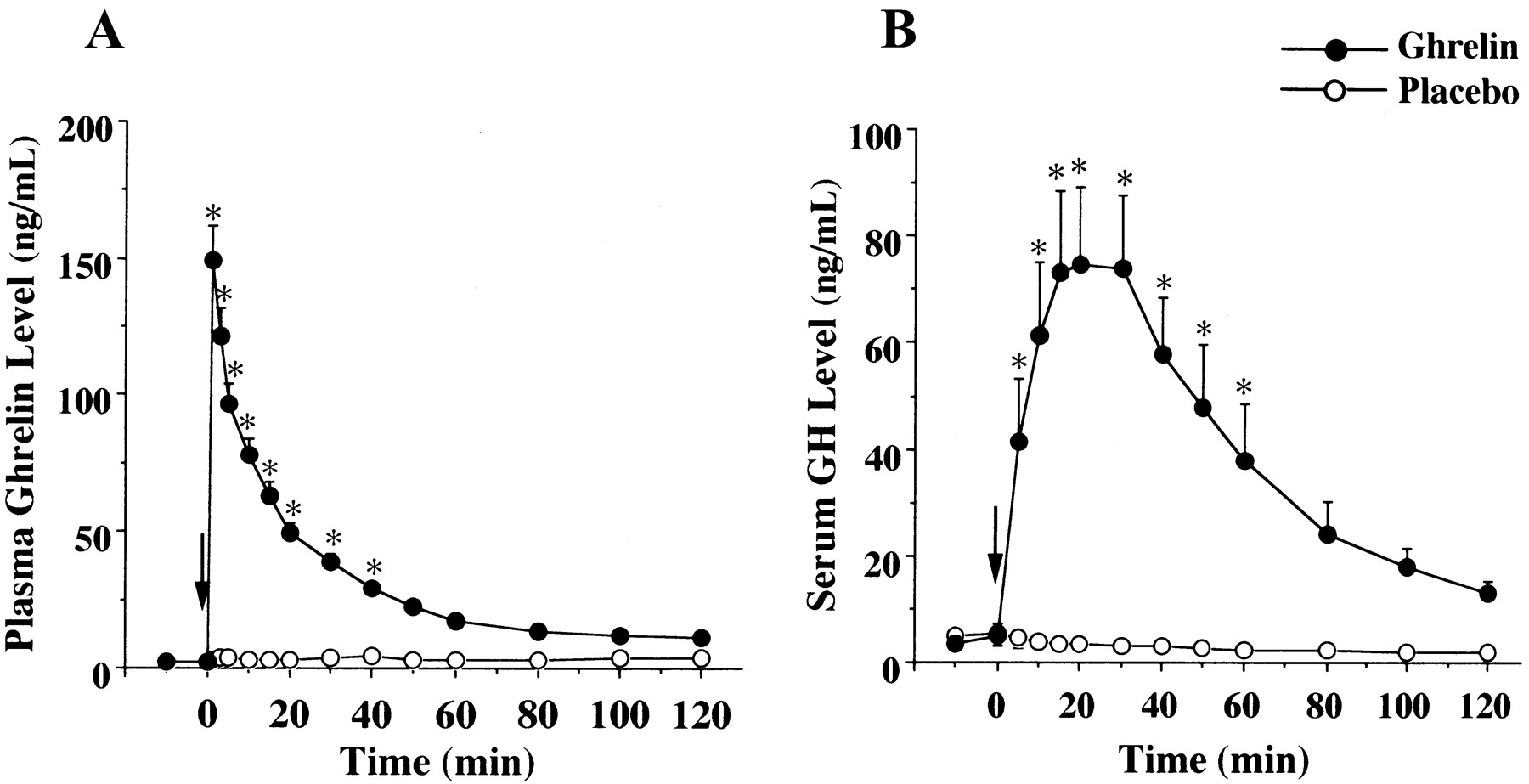


(Ref. ^E2^)

A) Circulating ghrelin level after a single injection of synthetic human ghrelin (10μg/kg).

B) Effects of a single injection of synthetic human ghrelin (10μg/kg) on circulating growth hormone (GH).

1. **Vasodilation and increase cardiac output** (Ref. ^E2^)


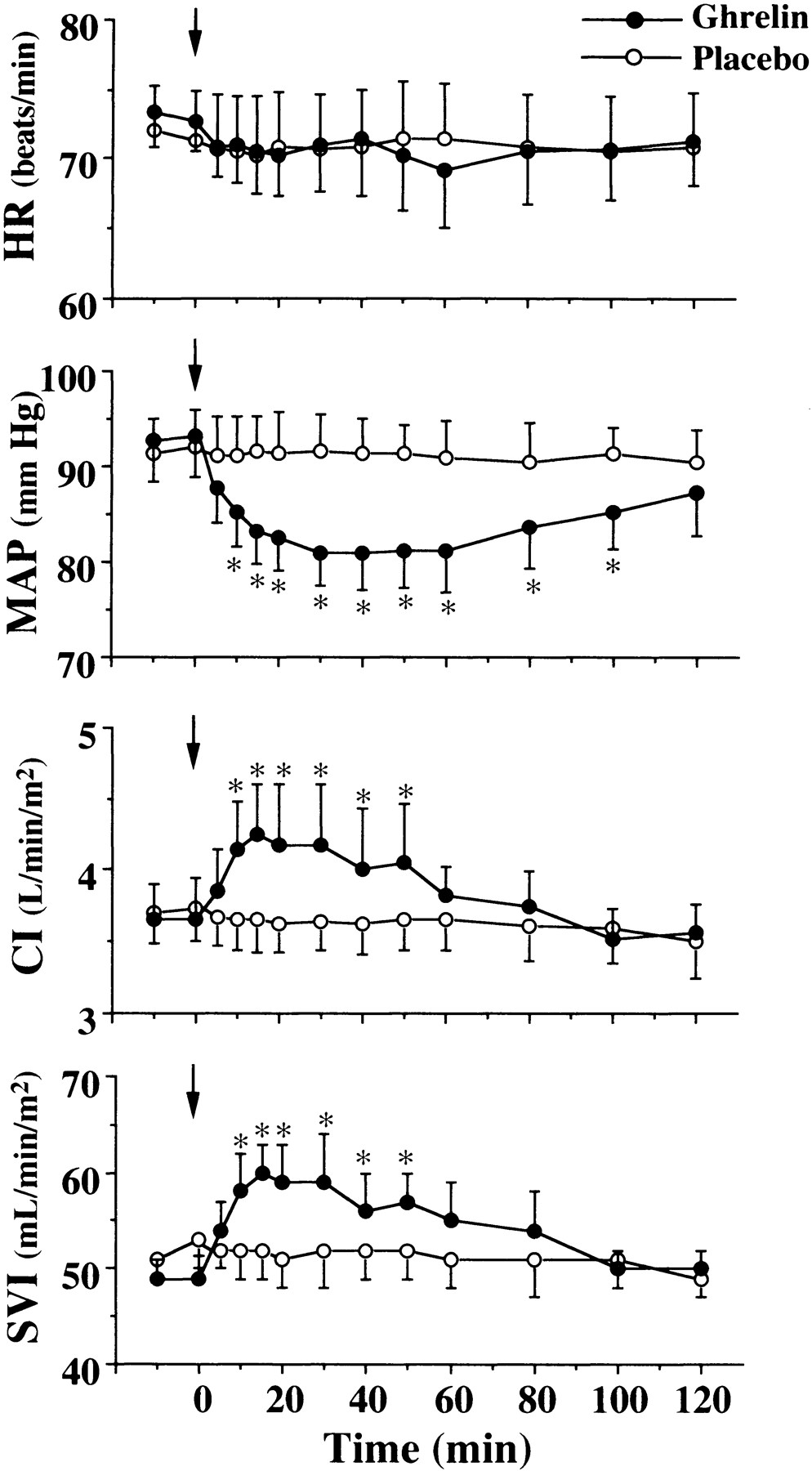


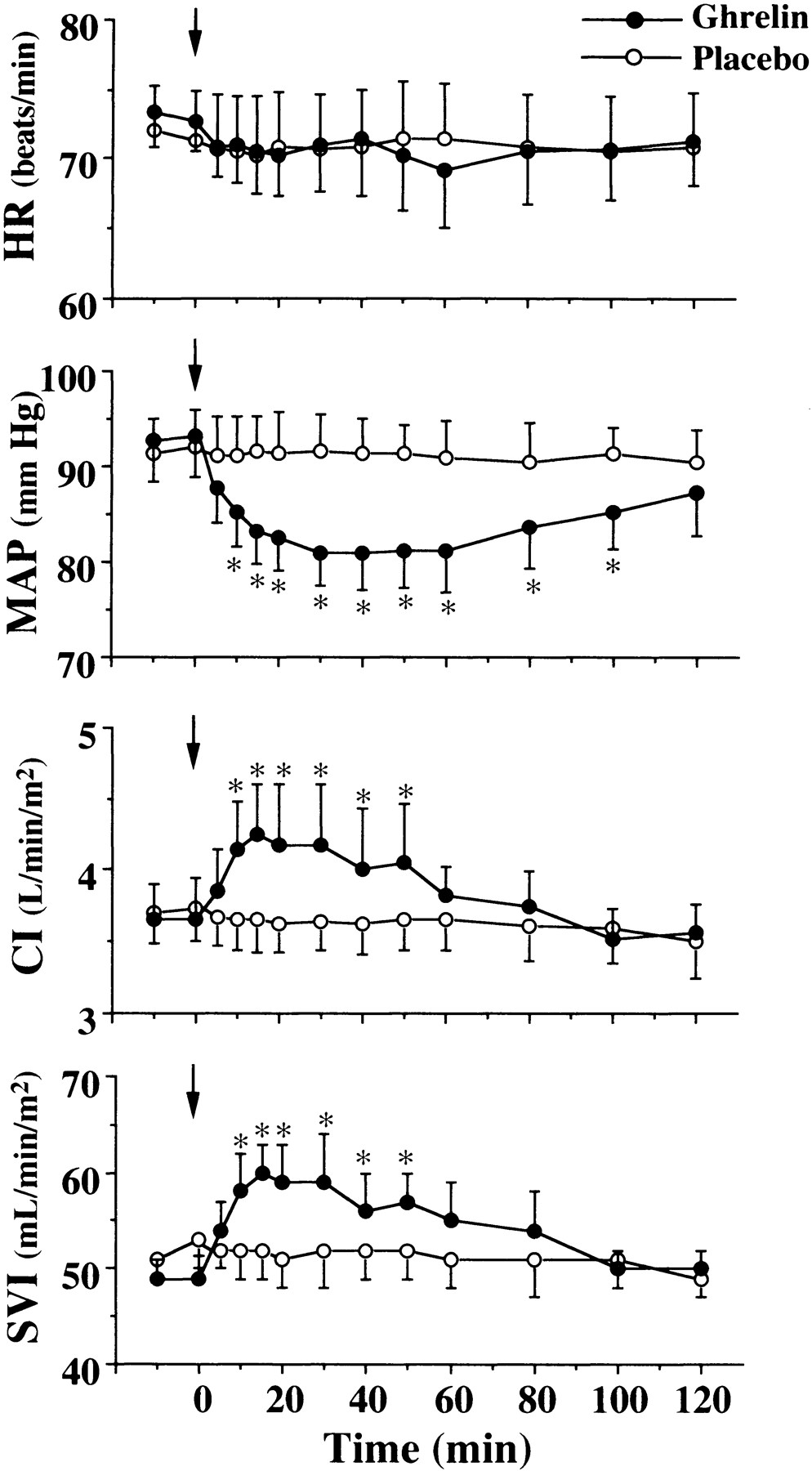


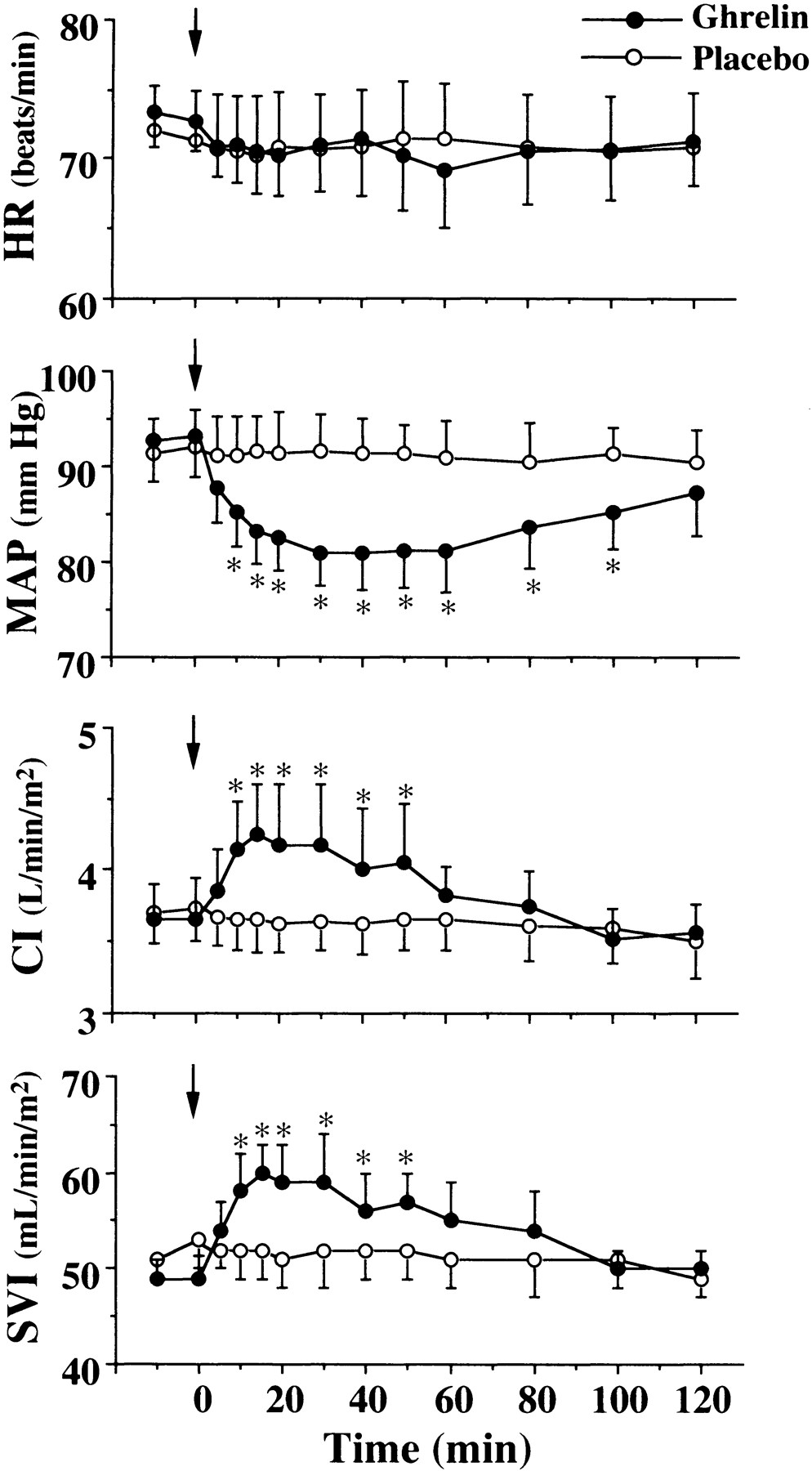

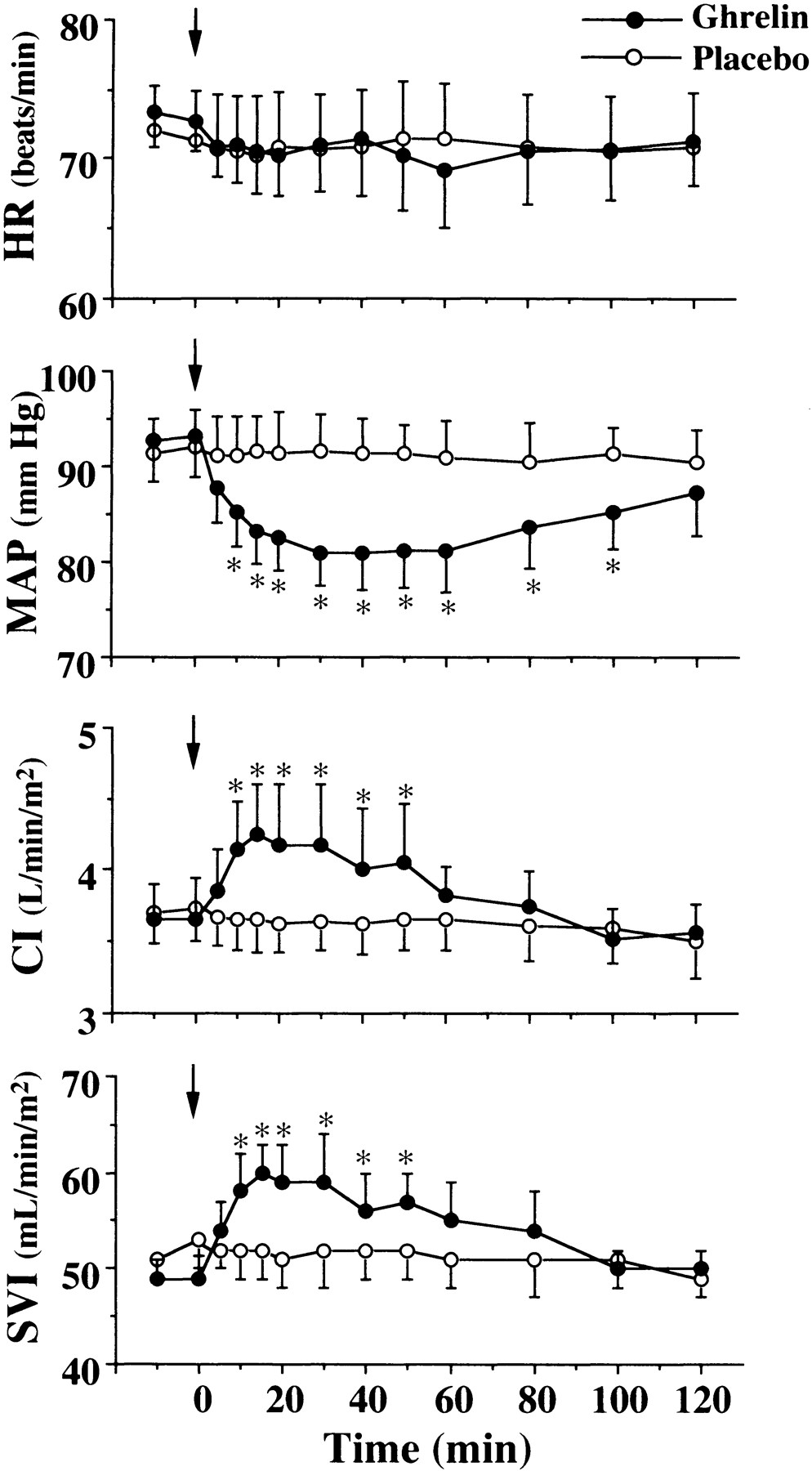


Effects of a single injection of synthetic human ghrelin (10μg/kg) on mean arterial pressure (MAP), cardiac index (CI). (Ref. ^E2^)

1. **Attenuation of sympathetic nerve activity** (Ref. ^E2, 4, 5^)
2. **Inhibition of apoptosis in cardiomyocytes and endothelial cells** (Ref. ^E6^)
3. **Stimulation of food intake** (Ref. ^E7, 8^)
4. **Induction of positive energy balance (**Ref. ^E9^)

Ghrelin has the physiological functions from (b) to (f) through GH-independent mechanisms.

**Ghrelin treatment of COPD**

In advanced COPD, not only the deterioration of the pulmonary function but also the poor nutritional condition, i.e. pulmonary cachexia occurs. Cachexia is an independent risk factor for death in such patients. The improvements of both pulmonary function and poor nutritional condition should be needed for the treatment of COPD. In cachectic patients with COPD, endogenous ghrelin increased to compensate for the cachectic state (Ref. ^E10^), the increase of which may be still insufficient. Given the above physiological function of ghrelin, the replacement treatment by exogenous ghrelin raise the possibility that the respiratory muscle strength and leg strength as well as the energy metabolism may be improved through both GH-independent and dependent mechanisms.

**Previous clinical studies**

We administered ghrelin to 10 chronic heart failure patients for 3 weeks starting in 2000 after approval by the Ethics Committee, and investigated the safety and cardiac function- and energy metabolism-improving effects.

(a) Safety: Mild borborygmus occurred in 6 patients, and 2 patients complained of mild dry mouth, but no severe adverse drug reaction was observed, and all patients completed the scheduled protocol.

(b) Effect: It was demonstrated that ghrelin administration elevated the GH level, inhibited sympathetic nerve activity, improved cardiac function, food ingestion, and exercise tolerance, and corrected energy metabolism (Ref. ^E11^).

We administered ghrelin to 7 COPD patients for 3 weeks starting in 2003 after approval by the Ethics Committee, and performed a similar investigation.

(a) Safety: Mild hot flushes and sleepiness occurred in 3 patients and one patient complained of mild dry mouth, but no severe adverse drug reaction was observed, and all patients completed this protocol.

(b) Effect: It was demonstrated that ghrelin administration elevated the GH level, inhibited sympathetic nerve activity, promoted food ingestion, improved respiratory muscle strength and exercise tolerance, and corrected energy metabolism (Ref. ^E12^).

However, these were pilot studies, and reinvestigation by performing a randomized double-blind controlled study is necessary.

**II Objectives of the clinical trial**

The therapeutic effects of ghrelin on undernutrition (cachexia), which was shown by the previous animal and clinical studies (improvement of exercise tolerance and energy metabolism, promotion of food ingestion, and inhibition of sympathetic nerve activity), and the underlying mechanisms will be verified in cachectic patients with COPD by performing part of a multicenter, randomized, double-blind, placebo-controlled study.

**III Clinical trial team (multicenter trial)**

**1) Participating institutions**

i) Department of Cardiovascular Medicine, National Cerebral and Cardiovascular Center (NCVC); ii) Department of Respiratory Medicine, National Hospital Organization (NHO) Toneyama National Hospital; iii) Second Department of Internal Medicine, Nara Medical University; iv) Department of Neurology, Respirology, Endocrinology and Metabolism, Internal Medicine, Faculty of Medicine, University of Miyazaki; v) Department of Respiratory Medicine, Graduate School of Medicine,

Osaka City University

**2)** Ghrelin Study Group

**Study director**

NCVC, Research Institute, Deputy Center Director, **Kenji Kangawa**

**Chief researchers**

NHO Toneyama National Hospital, Department of Respiratory Medicine, Deputy Director, **Ryoji Maekura**; Nara Medical University, Second Department of Internal Medicine, Prof. **Hiroshi Kimura**; Faculty of Medicine, University of Miyazaki, Department of Neurology, Respirology, Endocrinology and Metabolism, Internal Medicine, Prof. **Masamitsu Nakazato**; Graduate School of Medicine, Osaka City University, Department of Respiratory Medicine, Associate Prof. **Kazuto Hirata**; NCVC, Research Institute, Department of Regenerative Medicine & Tissue Engineering, Director, **Noritoshi Nagaya**;

***Investigators***

NHO Toneyama National Hospital, Department of Respiratory Medicine, Chief physician, **Toru Hiraga**; NHO Toneyama National Hospital, Department of Respiratory Medicine, M. D. **Keisuke Miki**; Nara Medical University, Second Department of Internal Medicine, Assistant Prof. **Masanori Yoshikawa**; Nara Medical University, Second Department of Internal Medicine, Research Associate, **Atsuhiko Fukuoka**; Nara Medical University, Second Department of Internal Medicine, M. D. **Shinsuke Murakami**; Faculty of Medicine, University of Miyazaki, Department of Neurology, Respirology, Endocrinology and Metabolism, Internal Medicine, Research Associate **Junichi Ashitani**; Graduate School of Medicine, Osaka City University, Department of Respiratory Medicine, M. D. **Yoshitaka Tateishi**; NCVC, Department of Cardiovascular Medicine, Chief physician, **Norifumi Nakanishi**; NCVC, Department of Cardiovascular Medicine, M. D. **Hideo Oya**; NCVC, Research Institute, Department of Regenerative Medicine & Tissue Engineering, M. D. **Kohichi Tanaka**; NCVC, Department of Radiology and Nuclear Medicine, Director, **Hiroaki Naito**; NCVC, Department of Radiology and Nuclear Medicine, Chief engineer, **Sakashita Yoshiharu**.

**Person responsible for preparation**

NCVC, Department of Pharmacy, Manager, **Takeshi Kotake**

(The pharmacy department of each institution)

**Test drug controller**

NCVC, Department of Pharmacy, Director, **Hideki Morishita**

(The pharmacy department of each institution)

**3) Chairperson of the administrative committee**

NCVC, President, **Hitonobu Tomoike**

**4) Efficacy and safety committee**

NHO Kinki-Chuo Chest Medical Center, Clinical Research Center, Center director, **Kentaro Kimura**; Ikuwakai Memorial Hospital, Department of Respiratory Medicine, President, **Naotsugu Kurihara**; Sakai Hospital Kinki University Faculty of Medicine, Department of Respiratory Medicine, Prof. **Yukio Nagasaka**; NCVC, Research Institute, Emeritus Center director, **Hisayuki Matsuo**.

**5) Personal information controller**

NCVC, Department of Radiology and Nuclear Medicine, Director, **Hiroaki Naito**; NCVC, Research Institute, Department of Regenerative Medicine & Tissue Engineering, Director, **Noritoshi Nagaya**.

(Person in charge at each institution)

**6) Person responsible for test drug allocation**

Hamamatsu University School of Medicine, Emeritus Prof. **Mitsuyoshi Nakashima**.

**7) Data analyses, Statistics**

Japan Cardiovascular Research Foundation, Data center

**8) Secretariat**

NCVC, Research Institute, Department of Regenerative Medicine & Tissue Engineering, Director, **Noritoshi Nagaya**; NCVC, Research Institute, Department of Regenerative Medicine & Tissue Engineering, M. D. **Kohichi Tanaka**.

**Study director**

**Study director**

NCVC Research Institute

Kenji Kangawa

**Secretariat**

NCVC Research Institute

Department of Regenerative Medicine

Noritoshi Nagaya

Kohichi Tanaka

**Chairperson of the Administrative Committee** NCVC

Hitonobu Tomoike

**Chief researchers**

Ryoji Maekura

Hiroshi Kimura

Masamitsu Nakazato

Kazuto Hirata

Noritoshi Nagaya

**Test drug controller**

Pharmacy department of each institution

**Person responsible for preparation**

Pharmacy department of each institution

**Person responsible for test drug allocation**

　Mitsuyoshi Nakashima

**Data analyses**

Japan Cardiovascular Research Foundation, Data center

**Efficacy and safety committee**

　Kentaro Kimura

Naotsugu Kurihara

Yukio Nagasaka

Hisayuki Matsuo

Study director conducts the study based on the plan and work content specified in the study protocol and supervises the works related to the study. Study director ascertains necessary information to predict risks and secure safety and actually ensures sufficient safety until completion of the study, respects dignity and human right of individual subjects, and takes necessary actions to protect personal information. Study director reports information necessary to survey adverse events and secure appropriateness and reliability of the clinical study to the heads and Ethics Committees of the participating institutions. Study director ascertains presentations at academic meetings and publications related to this study in Japan and other countries until completion of the study.

**Chief researchers**

Chief researchers perform the clinical trial at the individual participating institutions and integrate work related to the study. They are responsible for the duty of Study director at the individual institutions.

**Investigators**

Investigators perform the trial based on the plan and work content specified in the study protocol. They are under obligation to protect lives, health, privacy, and dignity of subjects employing specialized knowledge and clinical experience necessary to properly execute the trial. They sufficiently explain items necessary to perform the trial to subjects and obtain written informed consent.

**Person responsible for preparation**

Person responsible for preparation is in charge of preparing the active drug and placebo necessary to perform the trial. The concrete preparation methods are separately described in the study protocol.

**Test drug controller**

Test drug controller manages the test drugs at the pharmacy department of each participating institution and the key code allocation table. Person responsible for test drug allocation distributes a copy of this allocation table by the clinical trial initiation. When an investigator informs new subject registration, Investigational drug controller confirms the subject code and records the date of key open, name of person who opens the key, and subject code in the allocation table, and opens the key code (ghrelin or placebo) for one case in the order of the case number in the table. The drug for subject will be prepared following this key code. Test drug controller is obliged not to open key codes before subject registration or tell the key code content to investigators or others after key opening.

**Chairperson of the administrative committee**

Chairperson of the administrative committee thoroughly enforces researchers to respect dignity and human right of subjects and protect personal information in the clinical trial to prevent ethical, legal respects opinions of the Ethics Committees, and social problems caused by this study. Chairperson of the administrative committee and decides on execution, permission or non-permission of continuation of the study, and other items necessary for the clinical study.

**Efficacy and safety committee**

Efficacy and safety committee appropriately evaluates adverse events. The committee manages the key code allocation table separately from Investigational drug controller and opens the key, as needed.

**Personal information controller**

Personal information controller is responsible for management and protection of personal information of subjects. When a patient is registered as a subject, the patient will be recorded in the collation table after assignment of an identification number. This table will be strictly managed by Hiroaki Naito, Director, Department of Radiology and Nuclear Medicine, NCVC, or a responsible person of each institution. Case reports labeled with identification numbers alone will be collectively managed strictly by Noritoshi Nagaya, Director, Department of Regenerative Medicine & Tissue Engineering, NCVC. The risk of personal information leak is markedly reduced by separately and strictly keeping the table and case reports.

**Person responsible for test drug allocation**

Person responsible for test drug allocation prepares the key code allocation table by the clinical study initiation. Subjects will be randomly allocated to active drug and placebo groups following this key code table. The table will be collected at the study completion to confirm appropriate conduct of the trial.

**Data analysis and statistics**

The key code table will be separately kept from the test drug controller and opened for data analysis after fixation of all data.

**Secretariat**

Investigators select patients who meet the inclusion criteria and do not conflict the exclusion criteria, and inform the secretariat with eligible patients for registration. The secretariat communicates with and coordinates the departments to smoothly progress the study.

**IV The clinical trial**

**1) Identification of test drugs**

(a) Control drug

One mL of 3.75% mannitol solution will be sealed into a vial.

(b) Investigational drug

Generic name: Synthetic human ghrelin

Dosage form: Synthetic human ghrelin 120μg will be dissolved in 2 mL of 3.75% mannitol solution and sealed into a vial.

Storage: -20°C under light-protected condition

(c) Production method and pyrogen test (Figure)

Synthetic human ghrelin synthesized by Peptide Institute Inc. will be purchased as bulk powder. Its purity will be confirmed at National Cerebral and Cardiovascular Center Research Institute, and a repurification process will be added as needed. At the Department of Pharmacy, National Cerebral and Cardiovascular Center, the substance will be weighed and dissolved to prepare 3.75% mannitol solution, and the solution will be sterilized by filtration and aliquoted into vials as preparations. The purity will be confirmed when the lot changes at National Cerebral and Cardiovascular Center Research Institute, and pyrogen, endotoxin, and bacterial culture tests will be performed by an external contract testing institution. The above process will also be applied when the bulk powder is purchased from another company.

(d) Dispensing method

After thawing the frozen investigational drug at room temperature, the amount to be used will be diluted with saline to a total volume of 10 mL.

**2) Purity of the test drug**

Human ghrelin is synthesized at about 99.4% purity.

**3) Stability of the test drug**

Synthetic human ghrelin (100 μg/ml) dissolved in 3.75% mannitol solution was distributed into polypropylene tubes at 50 ml per tube and kept standing at room temperature. Samples collected after 0, 2, 4, 6, 8, and 12 hours were subjected to reverse-phase high-pedformance liquid chromatography (HPLC), and the absorbance was measured at 210 nm. The absorbance at each time-point is presented, regarding the absorbance at the experiment initiation (0 hour) as 100% (no des-acylghrelin was detected).

| hr | 0 | 2 | 4 | 6 | 8 | 12 |
| --- | --- | --- | --- | --- | --- | --- |
| % | 100 | 90 | 95 | 94 | 94 | 90 |

The ghrelin preparation was stable for at least 12 hours at room temperature, suggesting that there is no practical problem.

**Synthesis and preparation of human ghrelin**

Peptide Institute Inc.

Synthesis of human ghrelin

1) Completely protected peptide is synthesized employing the Fmoc-synthetic method

(the side-chain hydroxyl group at Ser3 is protected with a trityl group (Trt)).

2) The Trt group at Ser3 is selectively eliminated using diluted trifluoroacetic acid (TFA) containing

additives, and an octanoyl group is introduced.

3) Complete protection is eliminated using a high-concentration TFA containing additives to obtain

crude peptide.

4) The crude peptide is purified employing reverse-phase high-pedformance liquid chromatography and

ion exchange chromatography.

5) The sample is then converted to acetate using exchange chromatography using Muromack as a carrier.

6) The target compound is obtained by chromatographic purification using SephadexLH-20 as a carrier.

Bulk powder of synthetic human ghrelin

NCVC

Research Institute

Purity measurement

|

Repurification (as needed)

Each pharmacy

department Weighed and dissolved in 3.75% mannitol

|

Sterilization using a sterilizing filter

|

Ampule preparation 　　　Preparation

| |

Macroscopic inspection of foreign bodies Freezing

External contract testing institution

Pyrogen test

Endotoxin test

Bacterial culture test

**V The clinical trial program**

This clinical study will be performed after approval by the chairperson of the administrative committee.

**1) Study design of the clinical trial**

**Study design**: a randomized, double-blind, placebo-controlled clinical trial

Patient enrollment and randomized allocation:

Investigators will select patients who meet the inclusion criteria and do not conflict the exclusion criteria, and inform the secretariat with eligible patients for registration. The person responsible for registration will promptly inquire the investigator of the content if there is an item to be confirmed, confirm eligibility of the patient, and randomly allocate the drugs.

**2) Subjects of the clinical study**

**Study patients**

A) **Trial Inclusion Criteria;** Patients who meet all conditions below:

a) Severe to very severe COPD (forced expiratory volume in one second

(FEV1)/forced vital capacity (FVC) of less than 70% and FEV1 percent predicted of less than 50%)

b) Underweight (body mass index (BMI) < 21 kg/m^2^)

c) Clinically stable and able to participate in pulmonary rehabilitation

d) Between 20 and 85 years old

e) Signed the agreement for participation in this study

B) **Trial Exclusion Criteria**; Participants were excluded for any of the following.

a) Malignant tumours

b) Active infection

c) Severe heart disease

d) Hepatic dysfunction (serum aspartate aminotransferase and alanine

aminotransferase levels at least twice the upper limit of normal)

e) Renal dysfunction (serum creatinine levels ≥ 2.0 mg/dl)

f) Asthma

g) Definitely or possibly pregnant

h) Change in drug regimen within 4 weeks before participation in this study

i) Judged to be unable to participate in this study by their physician.

**3) Pulmonary rehabilitation** (on an inpatient basis)

a) Education and instruction of patients and their families

b) Physical therapy: Conditioning (relaxation, flexibility training, instruction in breathing, and synchronization of motion)

c) Exercise training: Method of exercise training: Exercise training was conducted in 3 sets daily, every weekday for 3 weeks (i.e. 15 days) at high-intensity targets using electromechanically braked cycle ergometers. The initial exercise level of each set was set for 6 min at the work rate corresponding to 60% of the peak Vo_2_ achieved on the baseline cardiopulmonary exercise testing (CPET). As tolerated by the subject, the exercise duration was initially increased to 10 min. After that, the training work rate was increased by 5 W and then extended to the work rate corresponding to 80% of the baseline peak Vo_2_. If the subject found the set intolerable, it was reduced to its previous setting. Supplemental oxygen was used if necessary to maintain an oxygen saturation > 90% during exercise training.

(It is assumed that no patient will dropout from this program. Accordingly, the number of dropouts will not differ between the active drug and placebo groups even if exercise tolerance is improved by ghrelin, i.e., all subjects can equally obtain an improvement by exercise therapy, and ghrelin administration is expected to add an improvement.)

**4) Administration method**

The patients will be randomly divided into 2 groups: active drug (ghrelin (2 μg/kg) dissolved in 10 mL of saline) and placebo (10 mL of saline alone) treatment groups. The number of patients will be 30 in each group. The test drug will be intravenously administered taking 30 minutes twice a day (before breakfast and supper) for 3 weeks, and the effect will be investigated after completion of the administration.

**5) Other treatments**

Therapies (medication and oxygen therapy at home) other than treatment with the allocated drug will not be changed, as a rule. When an investigator judges that change or additional drug administration and treatment are necessary, the investigator will report it to the Study Director.

**6) Preparation of ghrelin and placebo vials and safety test**

The test drugs will be sealed into vials through an automatic aseptic operation at the pharmacy department. The safety test of injection was entrusted to Japan Food Research Laboratories (52-1 Motoyoyogi-cho, Shibuya-ku, Tokyo, JAPAN). Vials randomly selected from the prepared ones were subjected to endotoxin and pyrogen tests.

**7)** **Efficacy Assessments**

Efficacy assessments are to be performed before the administration; Week 3 after start of the administration.

**[**Before the administration and Week 3 after start of the administration**]**

1. Examination: Blood pressure, Heart rate, Auscultation

(b) Body height, Body weight, BMI

(c) Blood test (blood will be collected after resting for 15 minutes)

(d) Peripheral blood examination (including differential leukocyte count)

(e) Biochemical examination (Na, K, Cl, Ca, Mg, GOT, GPT, ALP, LDH, γ-GTP, TP, ALB, T-Bil, D-Bil, T-CHO, H-CHO, TG, Cre, BUN, UA, CK, FBS, HBA1c)

High-sensitive CRP

Hormone (insulin, GH, IGF-1, DHEAS, BNP)

Visceral protein (Prealbumin, Retinol binding protein, Transferrin)

Cytokine (TNF-α, IL-6)

* Peripheral blood test, biochemistry, and DHEAS and visceral protein measurement will be performed at Toneyama national Hospital.

* Other samples, i.e., hormones (insulin, GH, IGF-1, and BNP) excluding DHEAS, cytokines (TNF-α and IL-6), and high-sensitivity CRP will be collected to the center and collectively measured (multicenter trial).

1. Sympathetic nerve activity (Blood / urinary adrenaline, noradrenalin)

(Plasma and acidic accumulated urine will be used for measurement in blood and urine samples.)

* Samples for sympathetic nerve activity measurement will be collected to the center and collectively measured.

(g) Pulmonary function test (flow volume, spirometric evaluation, and DLco)

(h) Electrocardiogram continuously for 24 hours (Holter ECG)

(i) Cardiopulmonary exercise testing (CPET)

Analyzing expired gas, symptom-limited CPET will be conducted on an electrically braked cycle ergometer. Incremental testing, which consisted of 2-min increments to 10 W, will be performed. Subsequent constant-load testing, which was conducted at 70% of the maximal incremental work rate obtained in pre-treatment, will be performed to measure the exertional inspiratory capacity (IC) at pre-treatment and post-treatment with the same work rate. CPET will be done while breathing room air until subject exhaustion. Expired gas data will be measured breath-by-breath and collected as 30-s averages at rest, during exercise at 2-min intervals, and at end-exercise. In addition, the evaluation of dyspnea intensity (Borg scale) and arterial blood analyses will be done at rest and during the last 15 s of each exercise stage and at end-exercise. Arterial blood samples for blood gas analyses, plasma norepinephrine, and plasma lactate will be obtained.

Measurement parameters:  VO_2_ , VCO_2_, VE, VE max/MVV, VE/VO_2_, VE/VCO_2_, VD/VT, HR, BP, O_2_pulse (VO_2_/HR), Work rate, inspiratory capacity (IC), Borg scale, SpO_2_, blood gas analyses, plasma norepinephrine level, plasma lactate level

**[**Week 1 and 2 after start of the administration**]**

1. Examination: Blood pressure, Heart rate, Auscultation
2. Body height, Body weight, BMI
3. Blood test: Peripheral blood examination, Biochemical examination,

High-sensitive CRP

**[**The first day of the administration and Week 3 after start of the administration**]**

On these 2 days, blood will be collected before administration and 30, 60, and 120 minutes after administration to evaluate the time-course blood ghrelin and GH levels.

**8) Discontinuation of clinical trial**

The trial will be immediately discontinued when the following conditions are met:

(a) Notification of withdrawal from the trial by the patient

(b) Judgment by the investigator or subinvestigator

i) Development of an adverse event judged as resulting in discontinuation by the investigator or subinvestigator

ii) Conflict with the inclusion or exclusion criteria

* In case of i), the trial will be immediately discontinued and the adverse event will be treated.

**9) Schedule of the clinical trial from admission to discharge**

The duration of hospital stay will be about one month.

Week 0: Submission of written consent

Week 1: Examination before administration

Weeks 2-4: Ghrelin or placebo administration

End of Week 4: Examination after administration

Week 5: Discharge

**Schedule of the clinical trial**

| Date | / | / | / | / | / |
| --- | --- | --- | --- | --- | --- |
| Week | 0 | 1 | 2 | 3 | 4 |
| Informed consent production | ○ |  |  |  |  |
| Ghrelin administration |  |  |  |  |  |
| Examination: BP, HR |  | ○ | ○ | ○ | ○ |
| Body weight, Height , BMI |  | ○ | ○ | ○ | ○ |
| Blood drawing (20mL and serial measurement) |  | ○ |  |  | ○ |
| Pulmonary function test |  | ○ |  |  | ○ |
| Holter ECG |  | ○ |  |  | ○ |
| CPET |  | ○ |  |  | ○ |
| Blood drawing 10mL |  |  | ○ | ○ |  |

**10) Blinding**

The person responsible for test drug allocation will confirm and record non-identifiability of the appearances and packages of the active drug and placebo before allocation. The persons responsible for allocation and in charge of allocation will not disclose the allocation information they acquire through the work until key open. Labeling on the outer boxes containing the test drugs will follow the law, and the study name, number of vials, ‘for study use’, expiry date, and storage conditions will be described. The person responsible for test drug allocation will establish an allocated drug management procedure and record in and out.

**11) Disclosure of data to the patients**

When a patient requests, only data of the patient will be disclosed after the completion of all tests and key open.

**12) Expected period of the Clinical trial**

From the ethical committee approval in 2005 to June 2008.

**VI Adverse events**

1. **Actions taken to handle adverse events**

When an adverse event occurs in a patient, and the event is judged as requiring treatment by the investigator or subinvestigator, the best treatment will be provided to the patient. The investigator will promptly report it to the efficacy and safety committee. Since the investigator cannot judge whether the patient belongs to the active drug or placebo group at the time of the occurrence of the adverse event, the efficacy and safety committee will open the key code allocation table distributed before study initiation by the person responsible for test drug allocation to identify whether the patient is treated with the active drug or placebo, and judge the possibility of causal relationship between the adverse event and ghrelin. When the investigator or subinvestigator judges that continuation of the trial is difficult due to the adverse event, the trial will be discontinued, and the patient will be followed until recovery to the original condition. Regarding the laboratory test values, blood pressure, and pulse rate, values judged as abnormal with clinical significance by the investigator or subinvestigator will be handled as adverse events.

**2) Adverse event definition**

Adverse events represent all unfavorable events (including abnormal laboratory test values) which develop when the test drug is administered regardless of the presence or absence of causal relationship with the test drug. Adverse events for which causal relationship with the test drug cannot be ruled out will be handled as adverse drug reactions.

Among adverse events, a serious adverse event is defined as any experience of following outcomes:

1. Death.
2. Requires inpatient hospitalization or prolongation of existing hospitalization.
3. Results in a disability (Dysfunction interfere with daily life).
4. An immediate risk of death (i.e., “life threatening”)
5. A risk of disability (i.e., Important medical events that may not result in death, be

life-threatening or require hospitalization, and that, based upon appropriate medical judgment, may jeopardize the patient and may require intervention to prevent one of the outcomes listed above.

Adverse events meeting the following conditions other than serious events are defined as “significant adverse events”.

1. Markedly abnormal variation of laboratory test values
2. Events resulting in discontinuation of the trial

(c) Causal relationship with the test drug (judged as the 2 categories below)

i) No causal relationship: The reason for the development of the adverse event is clear and rules out the causal relationship.

ii) Causal relationship cannot be ruled out: The reason for the development of the adverse event is unclear and cannot rule out the causal relationship, or the reason can be judged as being due to the test drug.

The severity of adverse events will be classified and recorded as follows:

1. Mild: Events are usually transient and do not impair daily living activities.
2. Moderate: Events do not impair daily living activities but are of sufficient discomfort

(discomfort accompanies activities).

1. Severe: Events completely impair daily living activities (normal activities are difficult).

Serious adverse events will be handled as follows:

1. Investigators prepare a respirator and drugs for emergency, such as adrenaline and

noradrenaline, to treat test drug-induced anaphylactic shock.

1. Staff of each institution engaged in the trial will appropriately support the actions.
2. The investigator will open the emergency key of the patient as needed, and quickly inform it to

the head of the institution and the Ethics Committee of National Cerebral and Cardiovascular Center by submitting a serious adverse event report.

**VII Ethical conduct of the study**

1) The study will be conducted in conformity with the ethical principles of the Helsinki Declaration and the study protocol after examination and approval by the Ethics Committee of each institution.

2) **Merits and demerits of the subjects**

a) Merits

Improvement of exercise tolerance and energy metabolism by ghrelin may be expected in the active drug group. Pulmonary rehabilitation performed in both active drug and placebo groups may improve dyspnea and exercise tolerance.

b) Demerits

Improvement of exercise tolerance and energy metabolism by the drug cannot be expected in the placebo group. Since the above merit expected in the active drug treatment group has not been established, the patients will be involved as collaborators for the study.

3) **Consent**

a) Preparation of explanatory document

(1) Investigators will prepare explanatory document to obtain consent from subjects to

participate in the study. The document will be revised as needed. The explanatory document must contain the items presented in b).

(2) The prepared or revised explanatory document is to be approved by the Ethics Committee

of each institution before the use.

b) Descriptions in the explanatory document

The explanatory document is to contain the following items:

(1) The objective of the clinical study is research.

(2) Objective of the clinical study

(3) Study method (inclusion criteria of subjects)

(4) Planned period of subject participation in the study

(5) Planned number of subjects participating in the study

(6) Expected risks and inconveniences (When no merit is expected for subjects, it must be informed to the subjects.)

(7) The presence or absence of other treatment methods and expected important merits and risks of these when patients are selected for the subjects

(8) Compensation and treatment that subjects can receive when health damage related to the clinical trial occurs.

(9) Subjects participate in the clinical trial by their own will and can reject or withdraw the participation any time. They will not be handled disadvantageously or lose benefit that they should receive due to rejection or withdrawal of the participation.

(10) When information which may influence subjects’ will to continue participation is obtained, it will be promptly informed to subjects.

(11) Conditions and reasons to discontinue participation in the study

(12) The Ethics Committee can inspect the clinical study records, in which subject’s secrecy will be kept. Sign and sealing or signature in the consent form by subjects permits inspection of their clinical study records.

(13) Secrecy of subjects will be kept when the study results are published.

(14) Content of payment when subjects have to pay for an expense.

(15)Content of payment when subjects receive payment (agreement on calculation of payment)

(16) Name, occupation, and contact address of the investigator or subinvestigator

(17) Items obliged to subjects

c) Timing and method of obtaining consent

(1) Investigators explain the clinical trial using the explanatory document and obtain written consent from subjects by their own will after providing them with sufficient time to consider.

(2) Participation based on consent by legal representative is not admitted in this study.

(3) The investigator or subinvestigator who explained the study signs the consent form and fills in the date. When a clinical trial collaborator supplementary explained, the collaborator also signs and fills in the date.

(4) The investigators keep the written consents.

(5) The explanatory document and consent (copy) are handed over to the subjects.

**4)**  **Revision of explanatory document and consent form**

a) When new information which may be related to consent of subjects is obtained during the clinical study period, investigators or subinvestigators will promptly inform it to subjects already participating in the study, confirm their will to continue the participation, and record the communication with the subjects on the information and their will are recorded.

b) Investigators or subinvestigators will revise the explanatory document and consent form based on the information as needed, and acquire approval from the Ethics Committee.

c) The revision will be promptly explained using the revised explanatory document and consent form to subjects already participating in the study, and consent by their own will be obtained.

**5)**  **Compliance with study protocol**

Investigators and subinvestigators will perform the study in conformity to the study protocol.

**6)** **Revision of study protocol**

When the study protocol has to be inevitably revised while the study is progressing, investigators can revise the study protocol as needed. Approval by each Ethics Committee is to be obtained to revise the protocol.

**7)**  **Deviation from the study protocol**

a) Investigator and subinvestigators must not deviate from or change the study protocol without approval based on the prior examination by the Ethics Committee.

b) Investigators and subinvestigators can deviate from or change the study protocol without approval by the Ethics Committee of National Cerebral and Cardiovascular Center to urgently avoid risks of subjects or due to other inevitable medical reasons. The investigator or subinvestigator will promptly report the content and reason of the deviation or change and propose the revision if it is appropriate to the Ethics Committee and obtain approval.

c) The investigator or subinvestigator prepares ‘records of deviation from the study protocol’ describing the contents and reasons of all deviations and changes.

**8)**  **Direct inspection of original documents**

In the survey to confirm proper conduct of the clinical study and reliability of data by a person approved by the Ethics Committee, investigators provide all clinical study-related records including the original documents for direct inspection.

**9)**  **Items related to human right of subjects**

a) Protection of subjects’ privacy is sufficiently considered on publication of the study results and handling of raw data related to the study and consent.

b) Information concerning privacy of subjects disclosed to the Ethics Committee on direct inspection of original documents will be protected by taking all appropriate preventive measures.

VIII **Discontinuation, suspension, or completion of clinical trial**

The criteria of discontinuation and suspension are as follows:

a) Occurrence of ethically or medically inevitable conditions, such as those required to secure subject’s safety

b) Loss of scientific validity to investigate the investigational drug

When the clinical trial is discontinued or suspended, the investigator will promptly notify it to the subject and take appropriate countermeasures. The study director has to report it to the Ethics Committee.

**IX Statistics Analyses**

**1)** **Statistics Analyses**

Statistical analysis of this clinical study will be performed by statisticians at the Data Center.

**2)** **Clinical effect**

The primary and secondary endpoints will be analyzed. Descriptive statistics (mean, standard deviation, median, and minimum and maximum values), 90% confidence interval, and 90% confidence interval of difference in the mean will be calculated.

Primary outcomes

1. Peak oxygen uptake (Vo_2_) measured by CPET

Secondary outcomes

1. Exertional dyspnea

2. Plasma norepinephrine levels

3. O_2_-pulse

4. Physiologic dead space /tidal volume ratio

5. Ventilatory equivalent for oxygen

6. Ventilatory equivalent for carbon dioxide

7. Inspiratory capacity

**X Audit of the clinical trial**

A person approved by the Ethics Committee will audit the clinical trial.

**XI Payment and compensation for health damage**

**1)** **Payment for study-related expenses**

Costs for the test drug and tests other than health care services provided by national health insurance will be covered by the Grant for Health Science.

1. **Compensation for health damage**

When health damage occurs in a subject due to the clinical trial, the subject can rapidly receive appropriate treatment. Compensation for this health damage is limited to treatment only.

**XII Intellectual property**

When a patent and economic profit related to the patent are produced as a result of the clinical study, the right belongs to the collaborative research institutions and persons executing the study including the country, research institutions including private companies, and researchers executing the study.

**XIII Items related to the Act on the Protection of Personal Information Held by Administrative Organs**

Personal information will be managed following the Act on the Protection of Personal Information Held by Administrative Organs.

1) Items of accumulated and analytical data are specified as those presented in Attachment 1 (case report form). These are the minimum necessary data for the study.

2) Data will be stored in 2 personal computers exclusive for management not connected to the hospital LAN. Each trial collaborator will use a single copy of case report form, and filled case reports will be strictly kept in custody with lock. Case reports which became unnecessary will be shredded thorough a shredder and discarded.

3) The personal computers for data management will be managed using devices to protect from robbery, such as wire lock. The system will be managed by access control and user authentication.

4) Samples collected from subjects will be immediately made anonymous by labeling with patient numbers.

5) When a change in the purpose of the use of private information presented at the time of collecting the information is judged as exceeding the rationale range, re-agreement will be obtained.

Information of the patients will be handled using only anonymous patient identification numbers. The concrete management method is as follows: When a target patient is registered as a subject of this clinical study, the patient will be recorded in the collation table after assigning a patient identification number. This table will be strictly managed by the personal information controller. The table of patients at NCVC will be strictly managed by Hiroaki Naito, Director, Department of Radiology and Nuclear Medicine, NCVC, and those of patients at the other institutions by responsible persons of the individual institutions. Case reports labeled with identification numbers alone will be collectively managed strictly by Noritoshi Nagaya, Director, Department of Regenerative Medicine & Tissue Engineering, NCVC. The risk of personal information leak is markedly reduced by separately and strictly keeping the table and case reports.

**Contact information**

Department of Internal medicine, National Hospital Organization Toneyama National Hospital, 5-1-1 Toneyama, Toyonaka, Osaka, 560-8552, Japan.

Phone: +81-6-6853-2001, Fax: +81-6-6853-3127,

E-mail: [mikisuke@toneyama.go.jp](mailto:mikisuke@toneyama.go.jp) (Keisuke Miki)

E-mail: [maekurar@toneyama.hosp.go.jp](mailto:maekurar@toneyama.hosp.go.jp) (Ryoji Mekura)

**Appendix 1**

**"Clinical Trial of Ghrelin Administration in patients with Chronic Obstructive Pulmonary Disease"**

**The information sheet (including Multicenter trial)**

**[Options of cooperation for the study and freedom of withdrawal]**

This document explains the clinical trial of external supplementation of ‘ghrelin’, a hormone present in the body, to ask whether you will give consent to participate in the trial.

The physician in charge will explain the content of ‘Clinical Trial of Ghrelin Administration in patients with Chronic Obstructive Pulmonary Disease’ as simple as possible, but please read the explanatory document yourself. If there is any unclear or anxious point, do not hesitate to ask. It will be explained until you fully understand the content of the clinical trial.

After you fully understand the content of the clinical trial, you will be asked whether you will give consent to participate in the trial. If you want to take time to consider the explained content or talk over with your family, friends, and acquaintances because you cannot decide on by yourself, you can decide on afterward.

If you agree to participate in the trial, you will be asked to clearly show your will in a written form by signing the consent form, in addition to orally informing it to the physician in charge.

Please decide on giving consent to participation in this trial by your free will. You will never be forced. You will not receive any demerit even if you do not consent. You can withdraw consent any time after giving it without any demerit. When you withdraw consent, your blood samples and test findings will be discarded, and your medical records will not be used for research thereafter. However, discard of the study results may be impossible in some cases, such as that the study results have already been published in a report at the time of withdrawal.

**The purpose and nature of the research**

Many previous treatments for chronic obstructive pulmonary disease including pulmonary emphysema are symptomatic treatments, but the disease has recently been considered preventable and treatable as treatment has been standardized based on scientific basis. In addition, the influence of the disease on the whole body has recently been attracting attention, and the importance of nutritional and exercise therapies has been pointed out. ‘Ghrelin’ was discovered in the human and animal bodies as a new substance promoting growth hormone secretion in 1999. Growth hormone plays an important role in the development of the body and maintenance of nutrition by activating protein synthesis to develop muscle, and promoting carbohydrate and lipid metabolism. Ghrelin not only promotes growth hormone secretion but also inhibits excess sympathetic nerve activity and enhances appetite. Accordingly, ghrelin injection is expected to increase weakened respiratory and lower limb muscle strength and improve emaciation by improving nutritional condition. In addition, these effects are expected to improve quality of life.

**Study objective:** The objective of this study is to evaluate whether ghrelin injection strengthens muscle and improves nutrition and subsequently the quality of life in patients with weakened respiratory and lower limb muscle strength due to chronic obstructive pulmonary disease and those with poor nutrition due to severe disease conditions. Ghrelin will be administered after you are admitted. All patients will undergo concomitant pulmonary rehabilitation during hospital stay. Participation by about 60 patients with chronic obstructive pulmonary disease is planned for this trial.

**Study method:** Ghrelin (active drug) or saline (placebo) will be administered by drip infusion taking about 30 minutes twice a day (before breakfast and supper) for 3 weeks. To judge the effect of ghrelin, the following items will be performed before and after administration: 1) Survey of objective symptoms by interview, 2) venous blood sampling (about 20 mL), 3) urinalysis, 4) arterial blood sampling, 5) respiratory function test, 6) food intake measurement, 7) 6-minute walk test, 8) measurement of skeletal muscle, bone mass, and fat, 9) 24-hour electrocardiography, 10) cardiopulmonary exercise test, 11) grip strength measurement, and 12) respiratory muscle strength measurement. To investigate the development of adverse drug reactions during the administration period, venous blood will be collected (about 10 mL per sampling).

You will randomly undergo treatment with ghrelin (active drug) or saline. You and we who will administer the drug will not be informed of which drug is administered to you during the administration period. All evaluations will be performed under blinded conditions.

**[Disclosure of the study protocol]**

You can read the study protocol if you wish.

**[Expected risks and countermeasures]**

Ghrelin administration may induce growling of the abdomen and hot flushes, but no serious adverse drug reaction has been reported. Since high-dose administration may reduce blood pressure, ghrelin will be administered at a low dose. Since ghrelin promotes growth hormone secretion, increased growth hormone-induced edema and digestive symptoms may develop. When these symptoms appear, the dose will be reduced. When no improvement is obtained, administration will be discontinued. Since ghrelin sterilized as other commercial drugs will be used, risk of infection through ghrelin administration may be very low. When several blood samples are necessary, a needle will be indwelled in the first injected vein and blood will be collected through it. Thus, painful puncture will be performed only once. The pain of needle puncture is the same as that on usual venous blood sampling.

**[Merits and demerits for cooperators]**

Ghrelin (active drug) administration may strengthen respiratory and lower limb muscles and improve energy metabolism. Since ghrelin administration has not been established as a treatment method, direct benefit of administered patients may be small. However, both patients undergoing active drug and placebo treatments will perform concomitant pulmonary rehabilitation. Since the efficacy of pulmonary rehabilitation is widely admitted, it may reduce respiratory distress and improve exercise tolerance. In addition, the achievement of the study will contribute to the medical development in the future, leading to effective diagnosis, prevention, and treatment of patients suffering the disease.

When health damage occurs in subjects due to this trial, it will be rapidly and appropriately treated. Compensation for this health damage will be limited to treatment alone.

**[Cost allocation]**

Regarding the costs necessary for this clinical trial, patients will pay for the hospital charge and tests covered by national health insurance. Costs for the test drug (ghrelin) and tests other than health care services provided by national health insurance will be covered by research fund. Transportation expenses and reward money will not be paid.

**[Intellectual property]**

A patent may be produced as a result of the clinical study. The right belongs to the collaborative research institutions and persons who execute the study including the country and research institutions including private companies. Economic profit related to the patent may be produced, but you do not have right for it.

**[Ethical consideration]**

Adequacy of the content and execution of this study protocol has been examined and approved from the scientific and ethical viewpoints. In addition, when changes in the protocol and methods occur, changes will be examined, and the maximum consideration will be given to safety and human right.

**[Personal information protection]**

Personal information used in this study will be properly managed following the Act on the Protection of Personal Information Held by Administrative Organs. Your personal information used for this study will be strictly managed.

The content of examinations and test findings obtained in this trial have to be always accurate, for which the Ethics Committee may directly inspect the information, but related persons are obliged to keep secrecy, and information concerning your privacy, such as your name, will never be leaked. The outcomes of this study may be summarized and published in medical journals, but there is no concern of leakage of your private information, such as your name.

The clinical trial will be immediately discontinued any time throughout the period in the following conditions:

1. Your request to discontinue the trial.

2. Occurrence of an adverse event making continuation of the trial difficult (any unfavorable medical event, for example, adverse drug reaction)

3. Serious deviation from or conflict with the study protocol

4. The physician in charge for the trial judges that discontinuation of the trial is necessary.

5. When information (such as new information concerning safety) which may influence subjects’ will to continue participation in the trial is obtained, it will be promptly informed to subjects.

If you have a question concerning your right and others after receiving this explanation, please ask any time. When health damage suspected to be related to the trial occurs, please contact and consult us immediately.

Date:

(Study Staff)

Division

Name

(Signature or Typed Name ⋅ Seal thereon)

**Public contact**

5-1-1 Toneyama, Toyonaka, Osaka, 560-8552, Japan.

Department of Internal medicine, National Hospital

Organization Toneyama National Hospital,

Phone: +81-6-6853-2001, Fax: +81-6-6853-3127,

E-mail: [mikisuke@toneyama.go.jp](mailto:mikisuke@toneyama.go.jp) (Keisuke Miki)

E-mail: [maekurar@toneyama.hosp.go.jp](mailto:maekurar@toneyama.hosp.go.jp) (Ryoji Mekura)

If you understand the above and consent to participation in this trial, please sign the consent form on the next page to record that you gave consent.

**[Filling in and storage of consent form]**

You will sign one copy of the consent form. Please ensure that you sign the signature box by yourself. No seal is necessary. The signed consent form will be copied using a copy machine and handed to you with the explanatory document.

**Appendix 2**

**"Clinical Trial of Ghrelin Administration in patients with**

**Chronic Obstructive Pulmonary Disease"**

**The informed consent form**

To the President of National Hospital Organization Toneyama National Hospital

I (Study Staff) have explained the purpose, nature, safety, possible risks of this research, based on the Information Sheet.

Date:

(Study Staff)

Division

Name

(Signature or Typed Name ⋅ Seal thereon)

The purpose, nature, possible benefits, and possible risks of this research; "Clinical Trial of Ghrelin Administration in patients with Chronic Obstructive Pulmonary Disease (Study director: Kenji Kangawa) " has been explained to me using the Information Sheet. I understand it, and that I can withdraw from the study at any stage and that it will not affect my medical care, now or in the future. I was given a chance to ask questions and feel that my questions have been answered. I know that being in this study is my choice. My signature below indicates that I agree to be in this study.

• Optionality of cooperation for the study and freedom of withdrawal

• The purpose and nature of the research

• Disclosure of the study protocol

• Expected risks and countermeasures

• Merits and demerits for collaborators

• Cost allocation

• Intellectual property

• Ethical consideration

• Personal information protection

Date:

Name:

(Signature or Typed Name)

Address

Phone ( ) –

**Appendix 3**

**"Clinical Trial of Ghrelin Administration in patients with**

**Chronic Obstructive Pulmonary Disease"**

**Request form for handling samples (discard and changes)**

Dear the President of National Hospital Organization Toneyama National Hospital,

I consented to cooperate for the "Clinical Trial of Ghrelin Administration in patients with Chronic Obstructive Pulmonary Disease (Study director: Kenji Kangawa)" performed by your institution, but I will partially change the previous consent. So, please properly handle my samples and all accompanying information.

Note

□Please discard and delete all my samples and information obtained during the trial.

□Please discard and delete all my samples and information obtained after the trial.

□Please use the samples only for studies related to this trial.

□Please use the samples only for medical studies.

Date:

Name:

(Signature or Typed Name)

Address

Phone ( ) –

**(National Hospital Organization Toneyama National Hospital** **fills in below. A copy of this form will be sent to you after completion of discarding samples.)**

As Mr./Ms. (ID number ) requested, please forward the procedure to discard samples and delete all accompanying information collected for the trial.

Person responsible for the study protocol: (Signature or Typed Name)

Physician who explained consent:

Invalidation of the above personal information and consent was confirmed.

Date:

Personal information controller: (Signature or Typed Name)

Discard of the above samples was confirmed.

Date:

Person responsible for sample management: (Signature or Typed Name)

Deletion of the above information was confirmed.

Date:

Person responsible for analysis: (Signature or Typed Name)

Discard of samples of Mr./Ms. and deletion and invalidation of all accompanying information were confirmed.

Date:

Study director: (Signature or Typed Name)

**References**

E1. Kojima M, Hosoda H, Date Y, Nakazato M, Matsuo H, Kangawa K. Ghrelin is a

growth-hormone-releasing acylated peptide from stomach. *Nature* 1999;402:656-660.

E2. Nagaya N, Kojima M, Uematsu M, Yamagishi M, Hosoda H, Oya H, Hayashi Y, Kangawa

K. Hemodynamic and hormonal effects of human ghrelin in healthy volunteers. *Am J*

*Physiol Regul Integr Comp Physiol* 2001;280:R1483-R1487.

E3. Date Y, Kojima M, Hosoda H, Sawaguchi A, Mondal MS, Suganuma T, Matsukura S,

Kangawa K, Nakazato M. Ghrelin, a novel growth hormone-releasing acylated peptide, is

synthesized in a distinct endocrine cell type in the gastrointestinal tracts of rats and humans.

*Endocrinology* 2000;141:4255-4261.

E4. Matsumura K, Tsuchihashi T, Fujii K, Abe I, Iida M. Central ghrelin modulates sympathetic

activity in conscious rabbits. *Hypertension* 2002;40:694-699.

E5. Date Y, Nakazato M, Murakami N, Kojima M, Kangawa K, Matsukura S. Ghrelin acts in the

central nervous system to stimulate gastric acid secretion. *Biochem Biophys Res Commun*

2001;280:904-907.

E6. Baldanzi G, Filigheddu N, Cutrupi S, Catapano F, Bonissoni S, Fubini A, Malan D, Baj G,

Granata R, Broglio F, Papotti M, Surico N, Bussolino F, Isgaard J, Deghenghi R, Sinigaglia

F, Prat M, Muccioli G, Ghigo E, Graziani A. Ghrelin and des-acyl ghrelin inhibit cell death

in cardiomyocytes and endothelial cells through ERK1/2 and PI 3-kinase/AKT. *J Cell Biol*

2002;159:1029-1037.

E7. Nakazato M, Murakami N, Date Y, Kojima M, Matsuo H, Kangawa K, Matsukura S. A role

for ghrelin in the central regulation of feeding. *Nature* 2001;409:194-198.

E8. Wren AM, Small CJ, Ward HL, Murphy KG, Dakin CL, Taheri S, Kennedy AR, Roberts GH,

Morgan DG, Ghatei MA, Bloom SR. The novel hypothalamic peptide ghrelin stimulates

food intake and growth hormone secretion. *Endocrinology* 2000;141:4325-4328.

E9. Tschop M, Smiley DL, Heiman ML. Ghrelin induces adiposity in rodents. *Nature*

2000;407:908-913.

E10 Itoh T, Nagaya N, Yoshikawa, M, Fukuoka, A, Takenaka, H, Shimizu, Y, Haruta, Y, Oya, H, Yamagishi, M, Hosoda, H, Kangawa, K, Kimura, H. Elevated plasma ghrelin level in

underweight patients with chronic obstructive pulmonary disease. *Am J Respir Crit Care Med.* 2004; 170: 879-82.

E11. Nagaya N, Moriya J, Yasumura Y, Uematsu M, Ono F, Shimizu W, Ueno K, Kitakaze M,

Miyatake K, Kangawa K. Effects of ghrelin administration on left ventricular function,

exercise capacity, and muscle wasting in patients with chronic heart failure. *Circulation*

2004;110:3674-3679.

E12 Nagaya N, Itoh T, Murakami S, Oya H, Uematsu M, Miyatake K, Kangawa K.

Treatment of cachexia with ghrelin in patients with copd. *Chest* 2005; 128:

1187-1193.
