# Supplementary material for: Effects of Ghrelin Treatment on Exercise Capacity in Underweight COPD Patients: a substudy of a multicenter, randomized, double-blind, placebo-controlled trial of ghrelin treatment
Source: BMC Pulm Med. 2013 Jun 10;13:37. doi: 10.1186/1471-2466-13-37 (PMC3683541; doi:10.1186/1471-2466-13-37)
Supplement: Additional file 2 — Effects of Ghrelin Treatment on Exercise Capacity in Underweight COPD Patients: a substudy of a multicenter, randomized, double-blind, placebo-controlled trial of ghrelin treatment. [file 1471-2466-13-37-S2.doc]

**Physiological** **Effects of Ghrelin in Cachectic COPD: substudy of a multicenter, randomized, double-blind, placebo-controlled trial of ghrelin treatment**

Keisuke Miki, Ryoji Maekura, Noritoshi Nagaya, Seigo Kitada, Mari Miki, Kenji Yoshimura, Yoshitaka Tateishi, Masaharu Motone, Toru Hiraga, Masahide Mori, Kenji Kangawa.

**Supplementary Results**

**Supplementary Results**

| Table E1.Peak exercise parameters at pre-treatment for the protocol with a ramp rate of 5 W/min and the one with 2-min increments to 10 W (n =20) | | |
| --- | --- | --- |
|  | Ramp rate of 5 W/min | 2-min increments to 10 W |
| Dyspnea, Borg scale | 6.9 (1.5) | 7. 3(1.5) |
| Combino_2_, ml/kg/min | 12.5 (3.4) | 13.3 (3.3) |
| CombinE, L/min | 28.8 (7.1) | 30.6 (6.2) |
| CombinE /Combino_2_ | 51.1 (12.5) | 67.6 (12.5) * |
| Combino_2_/HR, ml/beats | 5.1 (1.3) | 5.4 (1.3) |
| CombinE / indirect MVV, % | 103.5 (26.1) | 110.6 (26.6) |

Definitions of abbreviations: CombinE = minute ventilation; Combino_2_ = oxygen uptake; HR = heart rate; MVV = maximum voluntary ventilation. Data are presented as means (SD). * p < 0.001: between the protocol with a the ramp rate of 5 W/min and the one with 2-min increments to 10 W.
